# European Reference Networks as core health structures where referring genetic newborn screening positive infants: an innovative operational research framework

**DOI:** 10.3389/fpubh.2026.1822461

**Published:** 2026-06-10

**Authors:** Fernanda Fortunato, Rita Selvatici, Jan Kirschner, Stefaan Sansen, Emanuele Agolini, Silvia Ottombrino, Enrico Bertini, Antonio Novelli, Moshe Einhorn, Leslie Matalonga, Sergi Beltran, Ivo G. Gut, Alberto M. Pereira, Olaf Hiort, Franz Schaefer, Alain Verloes, Ansgar W. Lohse, Arthur A. M. Wilde, Christine Bodemer, Guillaume Jondeau, Hélène Dollfus, Holm Graessner, Irene M. J. Mathijssen, Ikram L'khssim, Jean-Yves Blay, Luca Sangiorgi, Pierre Fenaux, María del Mar Mañú Pereira, Victoria Gutiérrez Valle, Marta Mosca, Nicoline Hoogerbrugge, Rene Wijnen, Teresinha Evangelista, Carla D'Angelo, Thomas O. F. Wagner, Wout F. J. Feitz, Ruth Ladenstein, Zoltan Dobai, Nico Wulffraat, Nicola Ruperto, Paloma Jara Vega, Cinzia Maria Bellettato, Maurizio Scarpa, Michela Onali, Sébile Tchaicha, Alexis Arzimanoglou, Jun Oh, Dominic Lenz, Birute Tumiene, Aldona Zygmunt, Alessandra Ferlini

**Affiliations:** 1 Unit of Medical Genetics, Department of Medical Sciences, University of Ferrara, Ferrara, Italy; 2 Department of Mother and Child, University Hospital S. Anna, Ferrara, Italy; 3 Department of Neuropediatric and Muscle Disorders, Medical Center, Faculty of Medicine, University of Freiburg, Freiburg, Germany; 4 Sanofi, Diegem, Belgium; 5 Laboratory of Medical Genetics, Translational Cytogenomics Research Unit & Research Unit of Neuromuscular Disorders, Bambino Gesù Children's Hospital, IRCCS, Rome, Italy; 6 Genoox, Tel Aviv, Israel; 7 Centro Nacional de Análisis Genómico (CNAG), Barcelona, Spain; 8 Department of Endocrinology and Metabolism, Amsterdam UMC, Location University of Amsterdam, Amsterdam, Netherlands; 9 Division of Pediatric Endocrinology and Diabetes, Department of Pediatric and Adolescent Medicine, University of Lübeck, Lübeck, Germany; 10 Center of Pediatric and Adolescent Medicine, University of Heidelberg, Heidelberg, Germany; 11 Department of Genetics, APHP-Robert Debré University Hospital, Université de Paris, Paris, France; 12 Department of Medicine, University Medical Center Hamburg-Eppendorf, Hamburg, Germany; 13 Department of Clinical Cardiology, Heart Center, Amsterdam University Medical Centers, University of Amsterdam, Amsterdam, Netherlands; 14 Department of Dermatology, Necker Enfants Malades Hospital, AP-HP Center-Paris University, Paris, France; 15 Hospital Bichat-Claude Bernard, APHP, Université Paris Cité, Paris, France; 16 Institut de Génétique Médicale d'Alsace, University Hospital of Strasbourg and Strasbourg University, Strasbourg, France; 17 Centre for Rare Diseases and Institute for Medical Genetics and Applied Genomics, University of Tübingen, Tübingen, Germany; 18 Department of Plastic and Reconstructive Surgery and Hand Surgery, Erasmus University Medical Center, Rotterdam, Netherlands; 19 Centre Léon Bérard, Université Claude Bernard Lyon I, and Center de Recherche en Cancérologie de Lyon, Lyon, France; 20 Department of Rare Skeletal Disorders, IRCCS Istituto Ortopedico Rizzoli, Bologna, Italy; 21 Saint Louis Hospital, Paris Cité University, Paris, France; 22 Group of Translational Research in Cancer and Blood disorders in Children, Rare Anemia Disorders Research Laboratory, Vall d'Hebron Institut de Recerca (VHIR), Barcelona, Spain; 23 Rheumatology Unit, Azienda Ospedaliero Universitaria Pisana and University of Pisa, Pisa, Italy; 24 Department of Human Genetics, Radboud University Medical Center, Nijmegen, Netherlands; 25 Department of Pediatric Surgery, Erasmus MC Sophia Children's Hospital, Rotterdam, Netherlands; 26 Groupe Hospitalier Pitié-Salpêtrière, Assistance Publique-Hôpitaux de Paris (APHP), Paris, France; 27 University Hospital Frankfurt, Frankfurt, Germany; 28 Department of Urology, Radboud University Medical Center Amalia Children's Hospital, Nijmegen, Netherlands; 29 Department of Pediatrics, St. Anna Children's Hospital and St Anna Children's Cancer Research Institute, Medical University of Vienna, Vienna, Austria; 30 Department of Pediatric Rheumatology and Immunology, Wilhelmina Children's Hospital, University Medical Center Utrecht, Utrecht University, Utrecht, Netherlands; 31 UOC Reumatologia e Malattie Autoinfiammatorie, IRCCS Istituto G. Gaslini, Genova, Italy; 32 Department of Pediatric Hepatology, La Paz University Hospital, Molecular Hepatology Group, La Paz Institute of Biomedical Research (IdiPAZ), Madrid, Spain; 33 Regional Coordinating Center for Rare Diseases, Udine University Hospital, Udine, Italy; 34 ERN EURO-NMD Patient Advisory Board, Paris, France; 35 Hospital Sant Joan de Déu, Barcelona, Spain; 36 Department of Pediatric Nephrology, Medical University Medical Center Hamburg-Eppendorf, Hamburg, Germany; 37 Division of Pediatric Neurology and Metabolic Medicine, Center for Child and Adolescent Medicine, Medical Faculty, University Hospital Heidelberg, Heidelberg University, Heidelberg, Germany; 38 Faculty of Medicine, Institute of Biomedical Sciences, Vilnius University, Vilnius, Lithuania; 39 Pfizer Inc., Collegeville, Pennsylvania, PA, United States

**Keywords:** European reference networks (ERNs), genomic newborn screening, healthcare pathways, operational workflow, rare genetic diseases, screen4care, treatable genetic disorders

## Abstract

Rare diseases (RDs), affecting fewer than 5 people per 10,000, present unique challenges to usual care pathways due to their unique characteristics: rarity and large number of disease entities, heterogeneous clinical manifestations and genetic causes, multisystemic involvement, and high complexity of diagnosis and treatment. This complexity often hampers the setting of appropriate pathways of care, which are not easily identifiable by patients and stakeholders. This ultimately leads to significant delays in diagnosis, lack of timely access to RD treatments and profound inequalities across countries. To overcome these difficulties, European Reference Networks (ERNs) were established in 2017 to facilitate patients' referral to expertise and excellent services, aiming to reduce disparities and expedite diagnosis, standard of care, and treatment for people living with rare diseases (PLWRDs). Since 72% of rare diseases are of genetic in origin and mostly affect children, genomic newborn screening (gNBS) offers a powerful tool to overcome diagnostic barriers by providing early and accurate genetic diagnoses for a wide range of treatable pediatric RDs. Several gNBS initiatives have been implemented across Europe and worldwide. Screen4Care (S4C) is an EU-IHI funded research project integrating gNBS with artificial intelligence (AI)-based tools to improve care for PLWRDs in the EU. The project will offer gNBS to up to 18,000 infants using a capture-based panel (TREAT-panel) targeting 245 genes associated with treatable genetic disorders [ClinicalTrials.gov NCT06549218]. Within this framework, an operational pipeline and a comprehensive step-by-step process in collaboration with ERNs were developed to refer gNBS-positive newborns to the appropriate ERN, ensuring timely access to optimal standards of care and available treatments. We suggest that this organisational and structured health model might be adopted by EU Member States (MS), as it provides a defined clinical framework for identifying newborns with RDs at birth and ensuring they receive the correct care, thereby promoting patient-centred and equitable disease management.

## Introduction

Rare diseases (RDs) comprise 6,000–7,000 highly heterogeneous conditions affecting approximately 30 million people in the European Union and up to 450 million worldwide (1). Defined in the EU as conditions affecting fewer than 5 in 10,000 individuals, RDs pose major challenges to standard care pathways due to their rarity, clinical complexity, multisystemic involvement, and diagnostic difficulty (2). As a result, patients often experience unequal access to diagnosis and treatment and a prolonged diagnostic odyssey, with an average delay of 4.7 years, negatively impacting disease management, access to therapies and clinical trials, and family planning (3). For these reasons, RDs represent a global health problem, and it has been unanimously and internationally agreed that reducing fragmentation in health offers will optimize the standard of care and will improve the quality of diagnostic services to people living with rare diseases (PLWRDs) (4).

Given that approximately 72% of RDs are genetic in origin and 70% have pediatric onset, genomic newborn screening (gNBS) represents a promising and scalable strategy to enable earlier diagnosis and timely intervention (5). Recent advances in high-throughput omics technologies, together with improved bioinformatics capabilities (6) and rapidly decreasing sequencing costs (7), are expanding the range of diseases that can be screened and have supported the successful implementation of genetic and genomic screening strategies worldwide, including the establishment of international newborn screening consortia.

In this context, the EU-IMI initiative launched the Screen4Care (S4C) project in 2021 (8), an international public–private–patient consortium supported by the European Union and EFPIA and involving 38 academic and industrial partners.

S4C integrates gNBS with AI–based approaches to accelerate RD diagnosis. In detail, the project plans to screen 18,000 European newborns using the TREAT gene panel, which includes 245 treatable genetic disorders (9) [ClinicalTrials.gov NCT06549218]. Six disease selection criteria—treatability, clinical validity, age at onset, disease severity, penetrance, and next-generation sequencing-based genetic feasibility—were applied to prioritize genes to be included in the TREAT-panel.

Symptomatic infants with negative TREAT-panel results will be offered whole genome sequencing (WGS) to identify gNBS-escaped rare diseases and novel gene–phenotype associations. In parallel, S4C develops AI-based tools leveraging electronic health records and digital symptom checkers to enable early patient identification and support referral pathways, with the overall aim of reducing diagnostic delays, optimizing healthcare resource utilization, and assessing the cost-effectiveness and ethical implications of diagnostic strategies.

To address the pan-European nature of RD challenges and ensure equitable access to specialized expertise, 24 European Reference Networks (ERNs) were established in 2017 under a common EU legal framework, each dedicated to specific groups of rare or low-prevalence diseases (Table 1). ERNs currently connect more than 300 hospitals and 1,600 healthcare providers (HCPs) across 25 European countries, fostering cross-border collaboration in clinical care, research, and knowledge exchange (10).

**Table 1:** List of the established European Reference Networks and their websites.

	List of networks
**Endo-ERN**	European Reference Network on rare endocrine conditions. https://endo-ern.eu/
**ERKNet**	European Reference Network on rare kidney diseases. https://www.erknet.org/
**ERN BOND**	European Reference Network on rare bone disorders. https://ernbond.eu/
**ERN CRANIO**	European Reference Network on rare craniofacial anomalies and ear, nose and throat (ENT) disorders. https://www.ern-cranio.eu/
**ERN EpiCARE**	European Reference Network on rare and complex epilepsies. https://epi-care.eu/
**ERN EURACAN**	European Reference Network on rare adult solid cancers. https://euracan.eu/
**ERN eUROGEN**	European Reference Network on rare urogenital diseases and complex conditions. https://eurogen-ern.eu/
**ERN EURO-NMD**	European Reference Network on rare neuromuscular diseases. https://ern-euro-nmd.eu/
**ERN GENTURIS**	European Reference Network on rare genetic tumour risk syndromes. https://www.genturis.eu/l=eng/home.html
**ERN GUARD-HEART**	European Reference Network on uncommon and rare diseases of the heart. https://guardheart.ern-net.eu/
**ERN PaedCan**	European Reference Network on paediatric cancer (haemato-oncology). https://paedcan.ern-net.eu/
**ERN RARE-LIVER**	European Reference Network on rare hepatological diseases. https://rare-liver.eu/
**ERN ReCONNET**	European Reference Network on rare connective tissue and musculoskeletal diseases. https://reconnet.ern-net.eu/
**ERN RITA**	European Reference Network on rare immunodeficiency, autoinflammatory, autoimmune, and paediatric rheumatic diseases. https://ern-rita.org/
**ERN TRANSPLANT-CHILD**	European Reference Network on transplantation in children. https://transplantchild.eu/
**ERN-EuroBloodNet**	European Reference Network on rare haematological diseases. https://eurobloodnet.eu/
**ERN-EYE**	European Reference Network on rare eye diseases. https://www.ern-eye.eu/
**ERN-ITHACA**	European Reference Network on rare malformation syndromes, intellectual and other neurodevelopmental disorders. https://ern-ithaca.eu/
**ERN-LUNG**	European Reference Network on rare respiratory diseases. https://ern-lung.eu/
**ERN-RND**	European Reference Network on rare neurological diseases. https://www.ern-rnd.eu/
**ERN-Skin**	European Reference Network on rare, complex, and undiagnosed skin disorders. https://ern-skin.eu/
**ERNICA**	European Reference Network on rare inherited and congenital (digestive and gastrointestinal) anomalies. https://www.ern-ernica.eu/
**MetabERN**	European Reference Network on hereditary metabolic disorders. https://metab.ern-net.eu/
**VASCERN**	European Reference Network on rare multisystemic vascular diseases. https://vascern.eu/

By leveraging cross-border collaboration and digital health solutions and telemedicine tools, ERNs facilitate the exchange of expertise, research, and clinical experience without requiring patients to travel.

One of the most outstanding telemedicine tools is the Clinical Patient Management System (CPMS) 2.0 a digital platform that enables virtual clinical consultations for complex cases between top-level experts across all ERNs (11).

## Materials and methods

### Study design and organizational health framework

The S4C project adopts an implementation-oriented, multicenter organizational framework aimed at integrating gNBS outcomes with established ERNs to ensure timely diagnosis, standard of care, and treatment for infants affected by RDs. The framework is designed to support the operational interaction between national healthcare systems, scientific societies, associated partners, other NBS initiatives [e.g., ICONS (12)], and ERNs.

### Study population

The TREAT-panel gNBS pilot studies are designed to encompass a target population of up to 18,000 infants, representing one of the largest EU cohorts studied in this context to date. Babies' recruitment will be on competitive bases, and the interventional, prospective, clinical trial has been registered on ClinicalTrial.gov (NCT06549218). The resulting dataset will be stored in the S4C platform and then migrated to European Genome-Phenome Archives (EGA) (13) and therefore made accessible for further use, reuse, or secondary use in other research contexts. Recruitment is currently ongoing (started the 3rd of December 2024).

This unprecedentedly large cohort of patients studied is expected to lead to the identification of a substantial number of critical cases, affected by one of the 245 rare diseases or disease genes explored by the TREAT-panel or identified by WGS. We have preliminary estimated the number of possible critical cases based on the frequencies of rare, very rare, and ultra-rare diseases as reported by Nguengang Wakap and colleagues (14) (see Results).

### Referral to ERN centers

Within this framework, the S4C project engages in collaborative interactions with health systems at local, national, and European levels to facilitate timely access to clinical patient pathways (CPWs).

The involvement of ERNs is essential to ensure access to disease-specific expertise and high-quality services for patients across all Member States, thereby addressing existing inequities in care.

**Figure 1:**
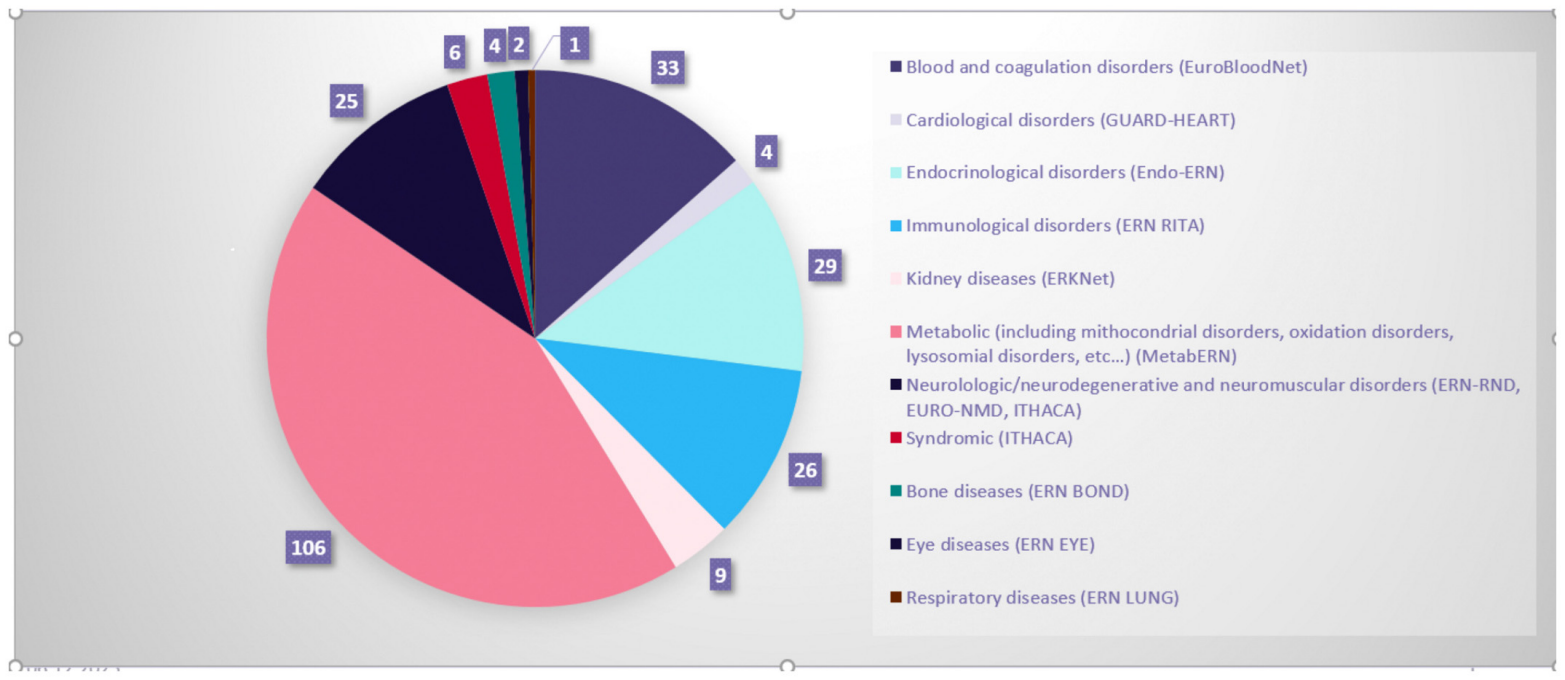
Distribution of genes included in the TREAT-panel by phenomic category and relative ERN.

Infants with positive TREAT-panel or WGS results will be referred to the appropriate ERN center with disease-specific expertise for diagnostic confirmation, comprehensive clinical evaluation, standard of care, and treatment. Based on the phenomic spectrum of the TREAT-panel, the full range of phenotypic categories associated with the screened genes are covered by ERNs (Figure 1 shows the distribution of genes included in the TREAT-panel by phenomic category and relative ERN). This pipeline also facilitates accurate data collection for disease registries and supports the implementation of the Clinical Patient Management System (CPMS) 2.0.

### Development of operational procedures for S4C–ERN interaction

To find consensus and design harmonized operational procedures, five structured online meetings were carried on involving both S4C partners and ERN Coordinators/Scientific Managers. These meetings provided a platform to identify the key components and define the operational framework, including referral pathways and implementation procedures. Written minutes were taken following each meeting, and critical issues raised, possible decisions, and areas of implementation were systematically discussed and agreed, to define the framework. Main discussion objectives of these meetings were defining modalities and strategies for addressing patients to the appropriate ERN centers, clarifying roles, responsibilities, and tasks of S4C and ERNs, and developing shared operational procedures that will serve as a standardized and transferable model for patient referral across different contexts and countries.

A semifinal draft of the operational procedures was than shared among all S4C partners and ERN Coordinators/Scientific Managers, enabling iterative final review, allowing to get a consensus.

### Data management and integration with ERN tools

Clinical and genomic data generated through the TREAT-panel and WGS are stored within the S4C platform and made accessible for further dissemination and use in other European contexts, in compliance with applicable data governance frameworks. The phenotypic spectrum associated with the TREAT-panel genes is managed through the relevant ERNs, facilitating accurate data collection for disease registries and supporting the implementation and use of the Clinical Patient Management System (CPMS) 2.0.

### Objectives of the referral framework

The overall objective of this methodological framework is to establish clear and efficient healthcare pathways for newborns affected by RDs in Europe, strengthening the connection between local and national healthcare providers and highly specialized expert centers within ERNs, and ensuring timely access to diagnosis, care, and treatment.

The study is conducted in accordance with applicable ethical standards and data protection regulations, including the General Data Protection Regulation (GDPR).

## Results

The work of members from S4C and ERNs has resulted in the formulation of a comprehensive and clearly delineated “step-by-step” process, which also specifies the responsibilities/tasks of S4C and ERNs. Moreover, the designation of “joint tasks” has been applied to those undertaken by S4C and ERNs in collaboration. In particular, the following tasks have been established, and the steps below describe an ideal referral process for positive infants.

### Infants recruitment modalities, samples collection, output analysis, data curation, and newborn screening tests technical and diagnostic validation

Infants are recruited at S4C birth centers in compliance with country-specific ethical approvals (Project protocol and related documents in Supplementary Data Sheet 1, 2). Recruitment follows what registered in ClinicalTrial.gov, is competitive and follows the rules to ensure an unbiased, representative sample collection across diverse populations. Indeed, different countries are included as providing neonates, via public hospital birth centers in France (1), Germany (3), Italy (5), Czech Republic (1), Greece (1), and Poland (1), supporting equality and equity in line with the heterogeneous ethnic composition of the European population.

S4C centers disseminate project information to couples/parents through dedicated videos, leaflets, and educational trainings at different pregnancy checkpoints (CKPTs): an “early CKPT” during the first half of pregnancy, a “major CKPT” at the end of pregnancy, and a “rescue CKPT” immediately after birth during dry blood spot (DBS) collection for routine metabolic NBS.

**Figure 2:**
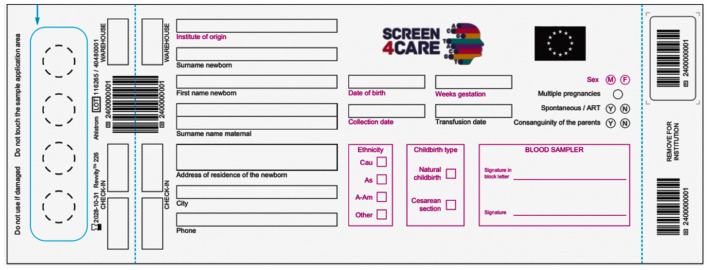
A project-specific DBS card with the S4C logo (S4C-DBS), designed by Revvity.

After obtaining informed consent, a S4C-specific DBS card (“S4C-DBS”, Figure 2) is distributed for collection of neonates' blood samples to avoid any interference between S4C and national screening programs. DBSs are pseudonymized at the birth centers and then shipped to the Bambino Gesù Children's Hospital (OPBG) which is the Hub for all procedures related to TREAT-panel sequencing, based on Next Generation Sequencing (NGS) strategies.

Raw sequencing data are transmitted via a secure, encrypted cloud platform to two research partners, Centro Nacional de Análisis Genómico (CNAG) in Barcelona and Genoox in Amsterdam. Results are accessible to consortium members through the RD-Connect GPAP and Genoox/CNAG Franklin platforms, with access restricted to S4C partners and subject to double-checking.

The scientific report, prepared and finalized by CNAG and Genoox, provide an overview of all identified variants within the TREAT-panel genes. Reports are then curated at the S4C validation Hubs (OPBG, University of Ferrara -UNIFE-, and Universitätsklinikum Freiburg -UKLFR-) to perform data curation and technical validation, particularly for copy number variations (CNVs) or complex loci. Curated reports are shared with the respective birth centers.

In case of infants with negative results, families are informed in written or digital form that no pathogenic or likely pathogenic variants were identified in the TREAT-panel (Negative report in Supplementary Data Sheet 3).

In positive cases, data curation is carried out in accordance with ACMG classification criteria. Varsome (15), ClinVar (16), and Franklin (17) were used as tools to accurately categorise the identified variants as pathogenic or likely pathogenic.

**Figure 3:**
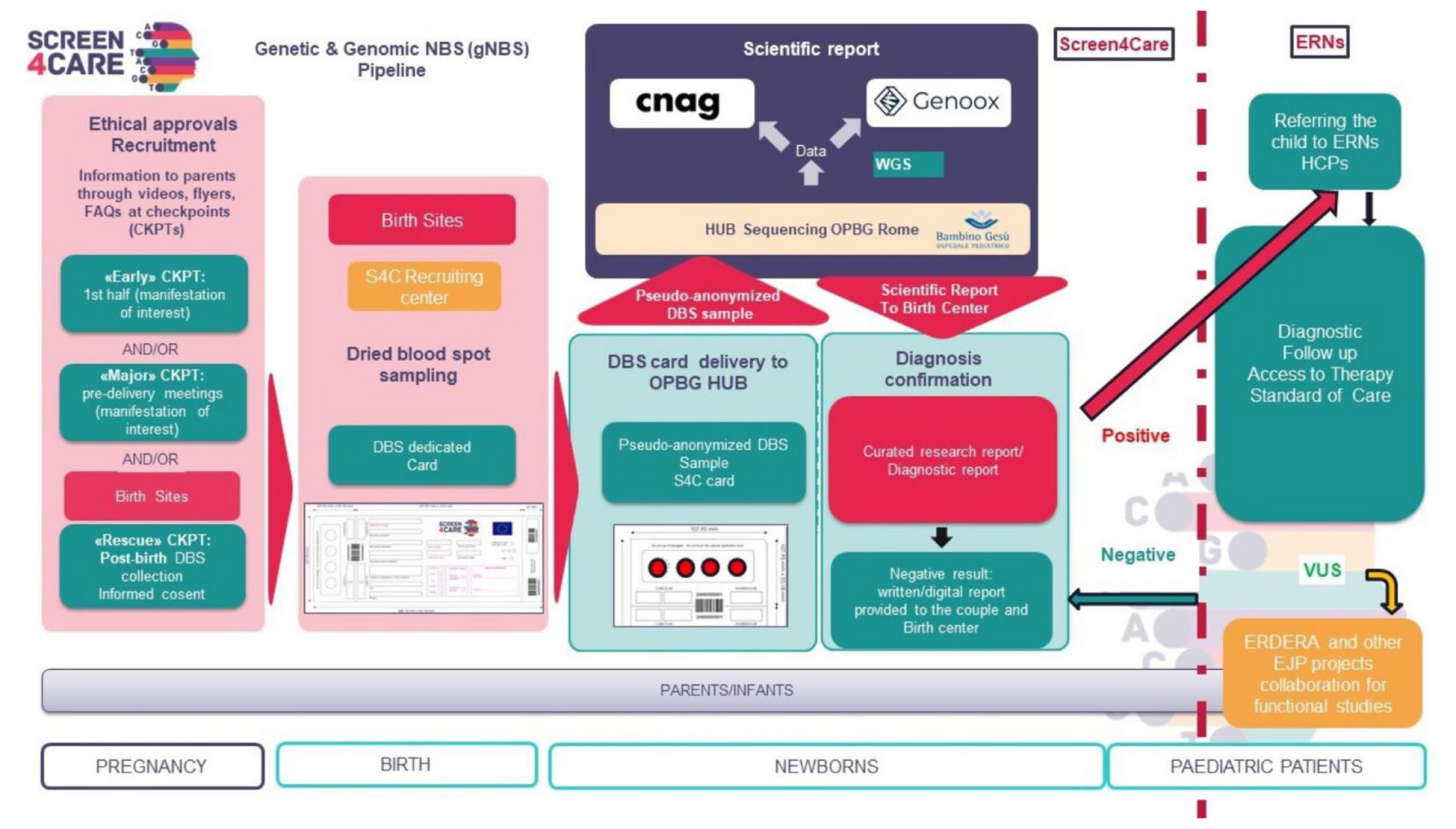
Schematic representation of S4C pipeline. The information to the parents is carried out through dedicated videos, flyers, and meetings during one or more pregnancy checkpoints (CKPTs): an early CKPT, a “major” checkpoint and a “rescue” checkpoint. If parents give consent to project' participation, a dedicated DBS card reporting the S4C logo (S4C-DBS) is distributed at S4C birth centers to collect blood samples from enrolled neonates. Samples are pseudonymized at S4C birth centers and shipped to the Hub Sequencing OPBG in Rome. The Hub Sequencing OPBG transmits the raw data of gNBS sequencing output via a dedicated, encrypted cloud platform (BaseSpace) to CNAG (Centro Nacional de Análisis Genómico) and Genoox, which are responsible to produce the scientific report. In case of negative result, a written/digital information is delivered to the family; WGS is proposed in case of early symptoms suggestive of a genetic disease within 12–24 months of life. In case of identification of a VUS, a pipeline to further study *in silico* or by functional studies of the VUS will be established at the HCP-ERN related, i.e., via European Rare Diseases Research Alliance (ERDERA) collaboration. In case of positive result, if technically validated, the family is referred to the closer specific HCP-ERN related. CKPTs, checkpoints; DBS, dried blood spot; NBS, newborn screening; OPBG, The Bambino Gesù Children's Hospital; CNAG, Centro Nacional de Análisis Genómico; WGS, Whole Genome Sequencing; VUS, Variant of Uncertain Significance; ERN, European Reference Network; S4C, Screen4Care.

Parents of positive babies are all contacted by S4C partners and birth centers and provided with a curated research report or a diagnostic report, if applicable, depending on the region/country ERN-related logistic (Supplementary Data Sheet 4), and then referred to the nearest phenotype- competent ERN for the standard of care (Schematic representation of S4C pipeline in Figure 3).

Variants of uncertain significance (VUS) are not reported, however, they are kept in the database and EGA to be eventually re-evaluated, as additional evidences become available, via European Rare Diseases Research Alliance (ERDERA) network collaboration. Indeed, a pipeline to further study “hot” VUSs using *in silico* or functional tools will be designed in collaboration with ERDERA (18).

### Referral of gNBS-positive infants to competent ERNs

Infants with positive gNBS results are referred to the phenotype-specific ERNs (HCPs and their clinical representatives) to receive standard of care from a multidisciplinary team. This process includes whole genetic counseling, family segregation and diagnostic-grade reporting (if needed), full clinical evaluation, and genotype-phenotype correlation. Following these steps, ERNs will provide as part of follow-up disease communication, explanation of clinical and reproductive implications, information and access, opportunities/modalities in currently available treatments, including orphan drugs.

This pipeline facilitates and accelerates the referral process, ensuring to gNBS-positive infants and their parents access to healthcare pathways.

The phenotypic competent ERN center is defined by two criteria: (a) *disease specificity*, infants referred to the ERN specific for the phenomic category; (b) *proximity*, the nearest ERN center, preferably in the infant's region of residence, as primary point of contact. Phenomic concept defines phenotypic groups composed by different signs and symptoms, and included in the TREAT-panel screened diseases.

Ultra-rare diseases may represent an issue, since the competent ERN might not be locally available. In these cases infants are referred to ERNs with recognized outstanding expertise in the specific condition using the CPMS 2.0 digital platform for clinical consultation to share patient information across ERNs, (see below for gNBS-CPMS synergies).

The ERNs are not only involved in delivering full standard of care but also in setting up diagnostic reports according to European Society of Human Genetics (ESHG) guidelines (19) and European Molecular Genetics Quality Network (EMQN) recommendations [since they do participate to all quality control assessment schemes, recommended to ensure quality grade to all diagnostic (20)].

**Figure 4:**
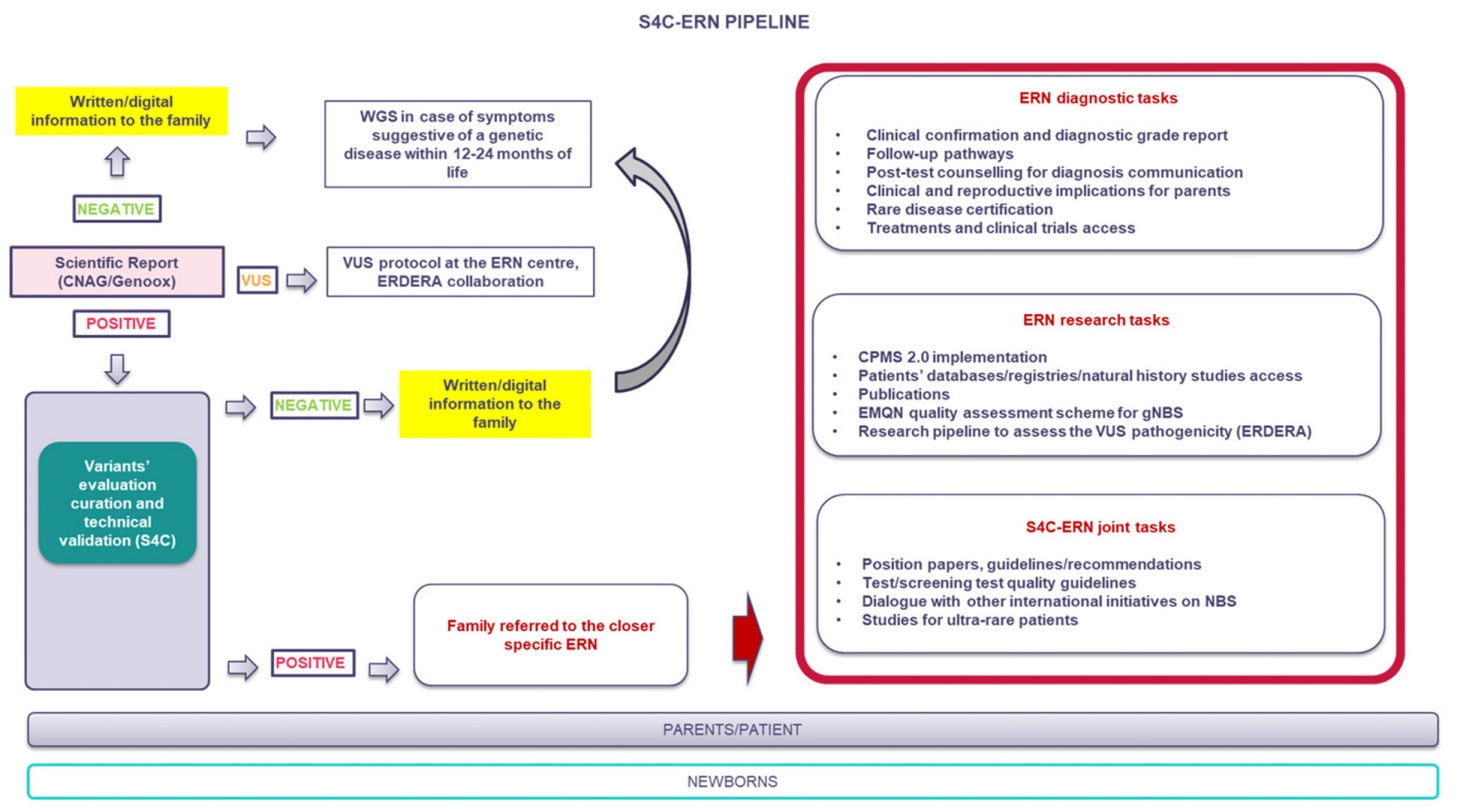
Schematic representation of S4C-ERN pipeline. The scientific report is produced by CNAG/Genoox. In case of negative result, a written/digital information is delivered to the family; WGS is proposed in case of early symptoms suggestive of a genetic disease within 12–24 months of life. In case of identification of a VUS, a pipeline to further study *in silico* or by functional studies of the VUS will be established at the HCP-ERN related, i.e., via ERDERA collaboration. In case of positive result, the family is referred to the closer specific HCP-ERN related. The ERN is responsible of diagnostic and research tasks. The term “joint tasks” has been applied to those tasks carried out in co-operation between the S4C and the ERNs. WGS, Whole Genome Sequencing; VUS, Variant of Uncertain Significance; ERN, European Reference Network; S4C, Screen4Care; CPMS, Clinical Patient Management System; gNBS, genomic newborn screening.

Finally ERNs coordinates the full range of multidisciplinary activities, as described on the specific ERNs website and EU recommendations, according to their own national organization, health mission, cross-cutting activities, and diagnostic working groups. As part of their duties, ERNs provides certification for rare diseases when applicable at the national level. Referral of gNBS-positive infants to ERNs ensures timely clinical evaluation and follow-up in accordance with standard-of-care guidelines, including available treatments, orphan drugs, and enrollment in novel clinical trials. A schematic representation of the S4C–ERN workflow is shown in Figure 4.

### gNBS and Clinical Patient Management System (CPMS) 2.0: a new synergy model

The Clinical Patient Management System (CPMS) 2.0 is a key tool implemented by ERNs to support active collaboration within and across the network and to facilitate data sharing (11, 21).

Within the S4C–ERN framework, CPMS 2.0 supports cross-border and multidisciplinary video meetings to discuss complex or ultra-rare cases and to share clinical expertise on the follow-up, diagnosis, and treatment of gNBS-positive infants. The platform also enables discussion of variants of uncertain significance (VUS) identified during genetic analysis.

In addition, by allowing patients' data to be made available for registries and databases, CPMS 2.0 contributes to the creation of a potential Electronic Health Record (EHR) database for rare disease patients, providing evidence and monitoring of clinical progress. Its use within the S4C–ERN workflow enhances the promotion of patient registries, natural history studies, and data sharing. gNBS-positive infants can also be included in ERN registries, increasing the availability of RD patients' data for research and clinical follow-up.

Therefore, early case sharing and rapid access to international expertise are enabled by CPMS 2.0, which provides a dedicated digital clinical pathway for ultra-rare diseases identified through newborn screening. This facilitates timely diagnosis and informed clinical decision-making from the outset.

The platform supports exceptional care by connecting multidisciplinary specialists, ensuring that each patient benefits from collective knowledge rather than isolated management. It also promotes excellence by spreading best practices, strengthening expertise across centres and improving consistency in care delivery.

Crucially, CPMS 2.0 reduces fragmentation in the management of ultra-rare diseases by integrating expertise, data and clinical pathways into a single, collaborative system.

### Estimated number of cases and feasibility implications

Based on the analysis of Orphanet dataset (14) we attempted to estimate some possible number figures of positive babies (18,000 newborns). We calculated to identify < 360 cases for rare (1–5/10.000), < 25 cases for very rare conditions (1–9/100.000), and < 12 cases for ultra-rare conditions (1/1.000.000) (see Supplementary Image 1).

This estimated gNBS positivity suggests that S4C would identify about 400 positive babies in total. Obviously this is a small but clinically very relevant proportion of newborns, heavily impacting on early diagnosis and access to treatment.

### Broader impact on child health

The S4C–ERN collaborative framework supports activities already included in the ERN mission, such as the development of guidelines and recommendations to define quality standards for gNBS and clinical follow-up of positive infants. It also facilitates the preparation of position papers on early diagnosis of rare diseases and the design of genetic screening quality assessment schemes in collaboration with the European Molecular Genetics Quality Network (EMQN) (20) and the European Society of Human Genetics (ESHG) (19).

This collaboration also promotes dialogue with other international NBS initiatives. A potential partner is ICoNS (The International Consortium on Newborn Sequencing), an alliance of genomic scientists and stakeholders working to implement newborn sequencing responsibly for the prediction of treatable diseases (12). Collaboration aims to facilitate the exchange of knowledge and perspectives among projects at different stages of implementation, including S4C, which is one of the funders of ICoNS.

Ultimately, the S4C–ERN collaboration supports the advancement of studies in ultra-rare patients by enabling coordinated access to a substantial number of cases, fostering research and improving clinical knowledge for rare diseases.

## Discussion

Genomic newborn screening (gNBS) raises several challenges. These include the interpretation of variants, data repository, management of carrier status, eventual medical costs for treatments, identification of late-onset disorders requiring follow-up, and the requirements for robust informed consent processes and clear communication with parents (22–25).

Among these challenges, the interpretation and reporting of variants of uncertain significance (VUS) represents one of the most critical and unsolved issues in genomics and consequently in gNBS. This is mainly due to the possible mild or even absent phenotype at birth in still asymptomatic babies, and the lack of information about VUSs is general mainly due to the scarse number of normal population genomic dataset. Obviously, communicating VUS to parents poses significant ethical and psychosocial challenges, including uncertainty, potential misinterpretation, and anxiety in the absence of an immediate opportunity for clinical intervention (26). Therefore, S4C representing a multicountry gNBS program will be helping to address the above critical issue and up to date (January 2026) it enrolled a total of about 7,500 newborns.

Ethical, legal, social, and data management considerations are therefore central to the design and implementation of gNBS programs, particularly in relation to data sharing across institutions and countries, long-term storage of genomic data, and harmonization with the General Data Protection Regulation (GDPR) requirements (27, 28).

S4C has obtained full ethical approval for gNBS using the TREAT-panel and WGS (Project protocol and related documents in Supplementary Data Sheet 1, 2). A transversal task, called Ethical, Legal and Safety Team (ELST) supported the preparation of Ethics Committee (EC) applications, with additional inputs from the Scientific Advisory Board (SAB) and Patients Advisory Board (PAB). Two separate EC applications were prepared for TREAT-panel and WGS, addressing inclusion and exclusion criteria, variants reporting policies, timing and modalities of result communication, sample storage, recruitment strategies, and parental information materials -including dedicated videos, flyers, and “*Frequently Asked Questions*” (FAQs)- (Supplementary Data Sheet 5 and Presentation 1).

For WGS, the reporting of incidental findings was explicitly defined according to current ESHG and ACMG guidelines (29, 30).

**Figure 5:**
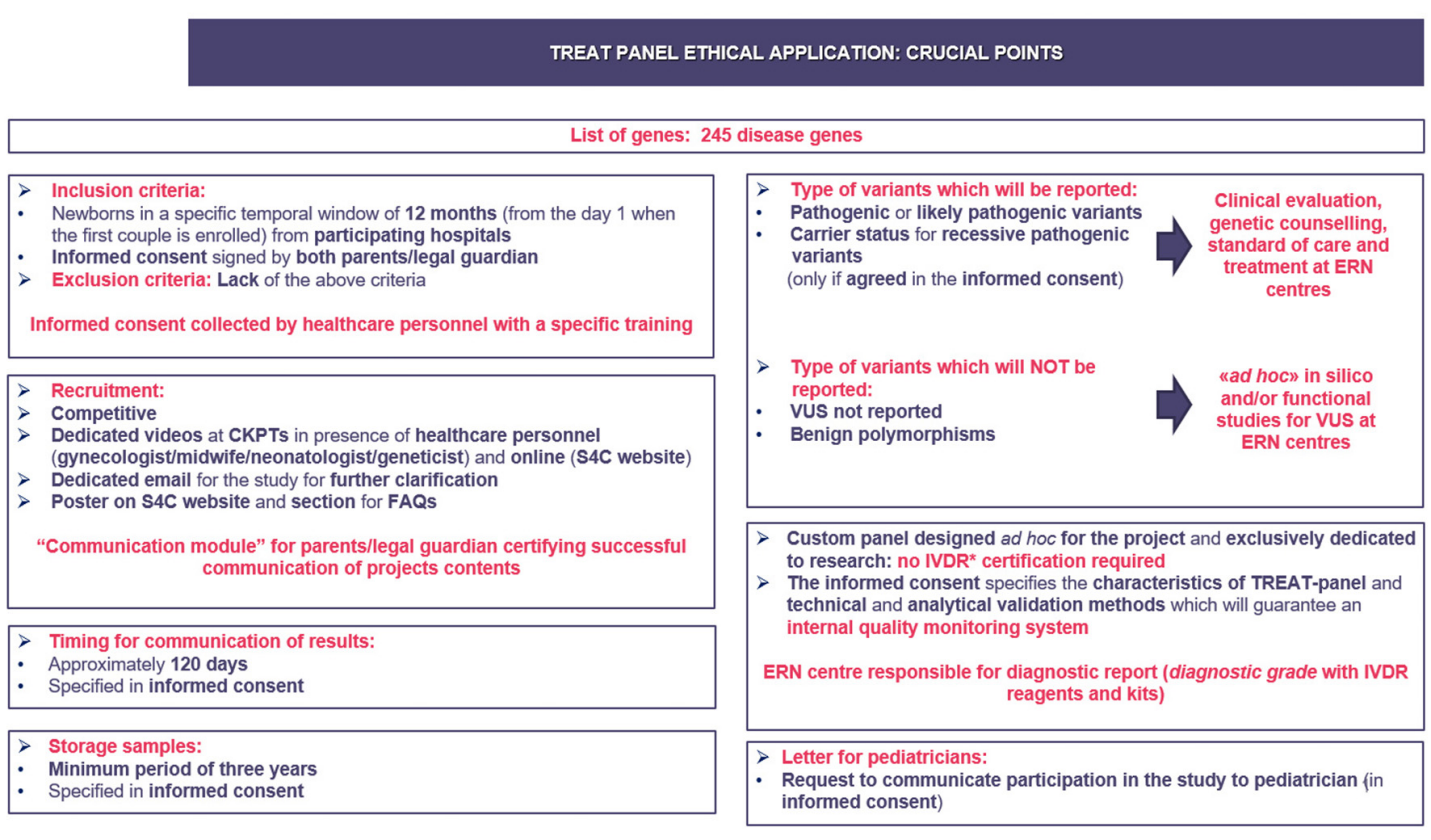
Detailed scheme of crucial points of ethical application for TREAT-panel. Overview of the key elements included in the TREAT-panel ethical application. The figure summarizes the main components of the ethical application, including inclusion and exclusion criteria, recruitment procedures, timing for communication of results, sample storage methods, and the types of variants to be reported or not reported. CKPT, checkpoints; ERN, European Reference Network; FAQ, Frequently Asked Question; IVDR, *In vitro* Diagnostic Regulation; VUS, Variants of Uncertain Significance.

**Figure 6:**
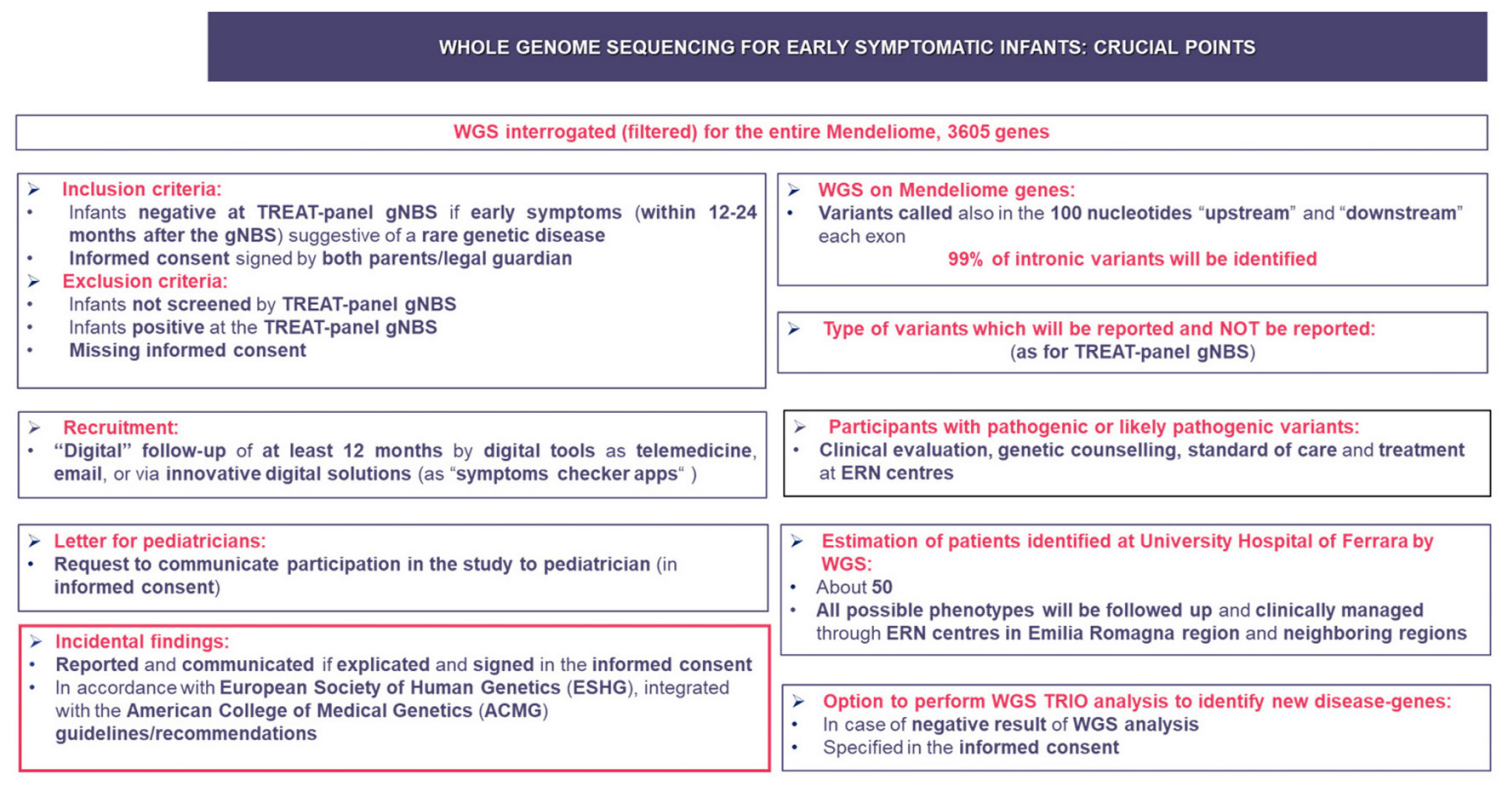
Detailed scheme of crucial points of ethical application for WGS. Overview of the key elements included in the ethical application for WGS. The figure highlights the main components of the ethical application, including inclusion and exclusion criteria, recruitment procedures, the types of variants to be reported and not reported, the management of incidental findings, and the estimated number of positive cases expected to be identified through WGS. ACMG, American College of Medical Genetics; ESHG, European Society of Human Genetics; ERN, European Reference Network; gNBS, Genetic newborn screening; WGS, Whole Genome Sequencing.

Finally, operational procedures for referral of positive infants to ERN centers, the main subject of this work, were cited and explicated in the ethical applications (Detailed scheme of crucial points of ethical application for TREAT-panel and WGS in Figures 5, 6). These elements were developed within the S4C structured governance framework designed to ensure both ethical compliance and operational feasibility across participating centers.

However, cross-border implementation of gNBS introduces additional challenges that remain only partially resolved in some current European countries' practice, particularly those regarding differences in national consent frameworks, variability in newborn screening policies, and heterogeneous thresholds for reporting and follow-up across healthcare systems, as previously described in the context of limited harmonization of screening programs (31). In addition, operational questions related to data governance and interoperability between national systems and European infrastructures remain critical for scalability, particularly in the context of cross-border genomic data sharing and the integration of ERNs (32, 33).

S4C is expected to generate a unique dataset integrating genomic and phenotypic information across multiple EU populations (34), the actual clinical and operational impact of such data will require systematic evaluation over time. Importantly, translating such large-scale genomic datasets into clinical practice requires the establishment of robust and standardized referral and care pathways across healthcare systems. Since we have 4M newborns in EU per year, we should be prepared to grant feasibility, high scaling, high performance, high throughput and parallelism strategies to the future gNBS if widely adopted in EU countries. Undoubtely, dedicated platforms and centers will be needed to face this challenge since scaling up requires considerations regarding sequencing capacity, bioinformatics pipelines, and output analysis. Nevertheless, the expected rate of positive neonates [we may foresee a 3% out of screened infants, as outlined in some published studies (35, 36)] is fully compatible with the current clinical infrastructures, as ERNs offer, making early and accurate the multidisciplinary care for newborns affected wih RDs.

Therefore, defining referral pipelines for gNBS-positive patients is outstanding and requires coordinated integration of care as well described in ERN frameworks for rare disease care (37). Achieving an appropriate balance between centralized expertise, local care provision, and digital health infrastructure remains a key implementation challenge in modern healthcare systems (38). The integration of ERNs into national healthcare systems, as supported by initiatives such as the Joint Action on Integration of ERNs into National Healthcare Systems (JARDIN) (39), represents a fundamental step toward strengthening these pathways. Our designed operational procedures and comprehensive “step-by-step” workflow will hopefully help in implementing and ensuring to RD patients a timely access to optimal standard of care and available treatments. Considering the triple mandate of ERNs -specialized care, research, and education- this collaboration may contribute to a model for RD clinical research and diagnostic pipelines. Ultimately, these operational procedures may help to identify a sustainable reference organizational model for gNBS and related health care pathways for RD patients that might be “adapted and/or adopted” by EU Member States' Health systems.

### Limitations

This study should be interpreted in light of several important limitations. The proposed framework is still in the early stages of implementation and there is no comprehensive prospective outcome data yet. Although recruitment for the S4C gNBS programme began on 3 December 2024, key performance indicators such as the number of infants recruited, referral rates to ERNs, turnaround times and diagnostic yield cannot yet be assessed robustly.

The manuscript primarily described the design of the gNBS and ERNs referral pathways within a complex, multi-country framework. Consequently, the generalisability and scalability of the proposed model across different national healthcare systems, regulatory environments and consent frameworks needs to be implemented.

Finally, the cross-border nature of the initiative introduces additional challenges relating to data governance, interoperability and harmonisation. These issues will be addressed by reinforcing the national impact of ERNs and their implementation. This will allow to better, systematically, and prospectively assess the clinical outcomes, the performance and the integration across ERNs and national health systems. While this framework provides a structured mechanism for referral to specialized care, several foreseeable operational constraints may arise in real-world implementation, including limited clinical capacity at the nearest ERN centre, absence of national ERN coverage for specific conditions, or situations in which families decline referral. These considerations highlight the need for flexible and contingency-based strategies within cross-border rare disease care pathways.

## Data Availability

The original contributions presented in the study are included in the article/supplementary material, further inquiries can be directed to the corresponding author.
