# Supplementary material for: European Reference Networks as core health structures where referring genetic newborn screening positive infants: an innovative operational research framework
Source: Front Public Health. 2026 Jun 10;14:1822461. doi: 10.3389/fpubh.2026.1822461 (PMC13292599; doi:10.3389/fpubh.2026.1822461)
Supplement: Supplementary file 1 [file Data_Sheet_1.pdf]

The European Commission's

Innovative Medicines Initiative (IMI) Screen for Care project (S4C)

STUDY PROTOCOL

SCREEN4CARE – Shortening the path to rare disease diagnosis by using newborn genetic screening and digital technologies

Genetic newborn screening for treatable genetic rare diseases through TREAT-panel/study TREAT-WGS within the Screen4Care project

The information contained in this document, is the property of the SCREEN4CARE consortium and is confidential. This information may not be disclosed, reproduced or distributed to anyone other than personnel directly involved in the conduct of the study and in response to a relevant Institutional Review Board/Independent Ethics Committee and Review by a Regulatory Authority as required by the applicable laws and regulations, without the written authorization of the consortium, except to the extent necessary to obtain informed consent from individuals who participate in the study. These restrictions will continue to apply after the study has closed.

Abbreviations

AI	Artificial Intelligence
CKPT	Checkpoint
CNAG	Centro Nacional de Analisis Genomico
DBS	Dried blood spots
DRKS	German Clinical Trials Register
EFPIA	European Federation of Pharmaceutical Industries and Associations
EHR	Electronic Health Records
EMA	European Medicines Agency
ERN	European Reference Network
FDA	Food and Drug Administration
gNBS	Genetic newborn screening
HCP	Health Care Provider
HPO	Human phenotype ontology
IMI	Innovative Medicine Initiative
ML	Machine learning
NBS	Newborn screening
NGS	Next generation sequencing
OPBG	Ospedale Pediatrico Bambino Gesù
RDs	Rare diseases
S4C	Screen4Care
VUS	Variants of uncertain significance
WGS	Whole genome sequencing
WP	Work package

Table of Contents

Sommario

1 Project summary 5

2 Responsibilities 6

2.1 **S4C Project Scientific Coordinator and Ferrara Team Lead** 6

2.2 S4C Work Package 3 Lead 6

2.3 Research Team 6

2.4 Project management 7

2.5 Duration and financing 7

2.6 Registration 7

3 Scientific Background 7

3.1 The Screen4Care project 7

3.2 Genetic newborn screening (gNBS) 9

4 Study goals 11

5 Study population 12

5.1 General 12

5.2 Inclusion and exclusion criteria 12

6 Methods and realisation 13

6.1 Sample collection and analysis 13

6.2 Follow-up for study participants 16

7 Assessment of benefits and risks 16

7.1 Benefits of participation 16

7.2 Risks of participation 17

a. Medical defensiveness 17

9 Ethical and legal aspects 18

9.1 Person responsible for data management and data collection 18

9.2 Information of participants and informed consent 18

9.3 Pseudonymisation 19

9.4 Data collection 19

9.5 Data transfer and data analysis 20

9.6 Data protection 20

9.7 Data storage 21

10 Appendices: 22

10.1 Appendix IA: General Screen4Care project overview 22

10.2 Appendix II: Criteria to select diseases for TREAT-panel 24

10.3 Appendix III: List of ERNs in which University Hospital of Ferrara, OPBG participate ….25

10.4 Appendix IV: Schematic representation of gNBS pipeline 27

10.5 Appendix V: Reference genes technical table and list of 244 genes included in the TREAT-panel 28

10.6 Appendix VII: Estimation of critical cases identifiable by TREAT-panel based on the frequency of rare, very rare and ultrarare diseases 36

10.7 Appendix VII: Phenomic categories associated to disease-genes included in TREAT-panel and related ERN 37

1 Project summary

This study is part of the EU research project “Screen4Care: Shortening the path to rare disease diagnosis by using newborn genetic screening and digital technologies” (www.screen4care.eu) and aims to investigate the use of next generation sequencing (NGS) technologies as part of newborn screening.

In parallel to established national newborn screening programs, we will offer research participants to perform NGS based genetic testing for a predefined number of genetic disorders. We aim to include 18000 participants in 6 participating countries (Italy, Germany, France, Greece, Poland and Czech Republic) . All participants will be tested for a set of TREATable diseases. Gene selection is always based on a predefined set of criteria. The TREAT-panel refers to genetic diseases with onset during childhood, for which there is an established treatment available.

Only pathogenic or likely pathogenic variants will be reported to participating families. Carrier status for monoallelic recessive pathogenic /likely pathogenic variants will be communicated if requested by the couple according to the informed consent. VUS will not be reported. In case of positive findings, full access to clinical examination and confirmatory genetic or metabolic tests will be ensured by the participating birth centers and clinical S4C partners through ERN collaboration. If presence of a genetic rare diseases is confirmed, families will be addressed to the ERN reference centres to initiate appropriate follow-up and treatment.

Families with negative genetic newborn screening tests will regularly be contacted for a follow-up period of at least 12 months. If infants will develop symptoms suggestive of a genetic disease, whole genome sequencing (WGS) of the entire Mendeliome will be freely offered as part of the research project. The WGS study protocol will be part of a separated, dedicated ethical application. Further, a technical validation TREAT-PANEL sub-study (involving a cohort of about additional 200 newborns TREAT-WGS cohort) is planned in Italy and is based on the use of WGS as a “technical validation tool” of the TREAT-panel, to test the accuracy of TREAT panel to identify both small variations and copy number variations (CNVs). In this study the genetic output will remain those of the TREAT panel study, therefore exclusively the 244 genes included in this panel will be “in silico” interrogated in newborns (TREAT-panel “in silico” with WGS backbone). No other information will be provided to the couples and all materials already presented in the TREAT panel study application will remain unchanged. A new informed consent is needed, since the newborn screening method will be WGS and not the TREAT panel.

2 Responsibilities

2.1 S4C Project Scientific Coordinator and Ferrara Team Lead

Prof. Alessandra Ferlini
Unit of Medical Genetics
S.Anna Ferrara University Hospital
Via Fossato di Mortara, 74
44122 Ferrara, Italy

2.2 S4C Work Package 3 Lead

Prof. Dr. Janbernd Kirschner
Department of Neuropediatrics and Muscle Disorders
Medical Center – University of Freiburg
Mathildenstr. 1, 79106 Freiburg, Germany
Janbernd.kirschner@uniklinik-freiburg.de

2.3 Research Team

Prof. Alessandra Ferlini
Scientific coordinator of Screen4Care
Unit of Medical Genetics, University of Ferrara, Ferrara, Italy
screen4care@unife.it

Prof. Dr. Enrico Silvio Bertini
Ospedale Pediatrico Bambino Gesù, Rome, Italy
enricosilvio.bertini@opbg.net

Dr. Antonio Novelli
Ospedale Pediatrico Bambino Gesù, Rome, Italy
antonio.novelli@opbg.net

Dr. Stefaan Sansen
Sanofi Aventis Group
stefaan.sansen@sanofi.com

Dr. Sergi Beltran
Centro Nacional de Análisis Genómico – Fundació Centre de Regulació Genòmica
sergi.beltran@cnag.crg.eu

Moshe Einhorn
Genoox
moshe@genoox.com

2.4 Project management

Dr. Kathrin Freyler
Department of Neuropediatrics and Muscle Disorders
Medical Center – University of Freiburg
christina.saier@uniklinik-freiburg.de

Dr Fernanda Fortunato
Unit of Medical Genetics
University of Ferrara, Italy
frtfnn@unife.it

2.5 Duration and financing

The study is funded as part of the Screen4Care project, which will run for a period of 6 years with a total budget of 25 million euro, provided by the IMI 2 JU, a joint undertaking of the European Union (EU) and the European Federation of Pharmaceutical Industries and Associations (EFPIA).

2.6 Registration

The study will be registered in ClinicalTrials.gov

3 Scientific Background

3.1 The Screen4Care project

This research project entitled "Screen4Care: Shortening the path to rare disease diagnosis by using newborn genetic screening and digital technologies" or Screen4Care (S4C), in short is a new EU Research Project and is an international public-private collaboration of 37 partners led by the University of Ferrara and including academic partners, industrial partners, small and medium-sized enterprises partners and EURORDIS. This ambitious project brings together experts with a wide range of expertise, including genetics, data management, ethics, and cybersecurity as well as the rare disease (RD) patient community.

S4C will last for a period of six years with a total budget of EUR 25 million provided by the Innovative Medicines Initiative (IMI 2 JU), a joint undertaking of the European Union and the European Federation of Pharmaceutical Industries and Associations (EFPIA). IMI is Europe's largest public-private initiative aiming to speed the development of better and safer medicines for patients.

In the EU alone, according to the Orphanet database (https://pubmed.ncbi.nlm.nih.gov/31527858/), 30 million persons, 3.5-6% of the general population, are affected by one of the 6,172 different rare diseases (RDs) of which 72% are genetic and 70% affect children. The path to diagnosis for people suffering from a RD is burdensome, often severely delayed by a diagnostic odyssey. People living with RDs often find themselves on a burdensome journey to diagnosis, enduring on average of eight years of inconclusive consultations and possible misdiagnoses, leading to ineffective treatments and inefficient healthcare resource utilisation. Lack of timely diagnosis affects disease management, family planning, identification of potential beneficial treatments and / or clinical trials. This unacceptable situation does not meet the concept of equity for EU citizens, and requires rapid, structured, and cost-effective corrective actions. The S4C consortium will leverage the genomic and digital advent to develop and pilot genetic newborn screening (gNBS) and AI-guided symptom recognition algorithms, while accounting for all relevant legal, regulatory and ethical considerations.

The project offers an innovative research approach to significantly shorten the time required for RD diagnosis and efficient intervention, through two central pillars: gNBS and digital technologies.

1. **Genetic newborn screening:** The project will drive newborn screening, using genetic testing and related advanced genomic technologies, which is anticipated to be an effective tool for early diagnosis considering that approximately 72% of RDs have a genetic cause and 70% of RD patients are children. In particular, gNBS, will adopt different strategies interrogating: I) currently treatable RDs (TREAT-panel-approach). In addition, whole genome sequencing (WGS) of the entire Mendeliome will be offered to early symptomatic patients enrolled in gNBS studies to identify known RDs, not included in the gene panel(s) and novel genes/phenotypes. Further, a short technical validation TREAT-PANEL sub-study (involving a cohort of about additional newborns TREAT-WGS cohort) is planned in Italy and is based on the use application of WGS as a “technical validation tool” of the TREAT-panel, to test the accuracy of TREAT panel to identify both small variations and copy number variations. In this study the genetic output will remain those of the TREAT panel study, therefore since exclusively the 244 genes included in this panel will be “in silico” interrogated in newborns (TREAT-panel “in silico” with WGS backbone). No other information will be provided to the couples and all materials already presented in the TREAT panel study application will remain unchanged. A new informed consent is needed, since the newborn screening method will be WGS and not the TREAT panel.
.
2. **AI-based tools:** The project will design and develop new Artificial Intelligence (AI) algorithms to identify patients at early disease onset via Electronic Health Records (EHR) and develop a repository of AI “symptom checkers” to help patients who are in the midst of their diagnosis journeys – both supporting symptom-based diagnosis later in life. Therefore, S4C aims to use the power of innovative digital solutions to shorten time to diagnosis via two routes: I) predictive

algorithms, leveraging the S4C data (federated) machine learning (ML) environment and embedded EHR systems; II) a repository of AI “symptom checkers”, which will be designed to facilitate self-diagnosis and/or to suggest referral pathways to physicians for diagnostic workup.

In addition, S4C aims to establish a digital infrastructure and ecosystem to engage patients, parents of newborns and caregivers as equal decision-makers in the diagnosis process. This project will allow continuous data collection and information exchange, contributing to the development of next-generation diagnostics and enabling physicians, patients and relatives to make informed decisions at an earlier stage. For a general project overview see **Appendix I** (**A & B**).

3.2 Genetic newborn screening (gNBS)

Expanded newborn screening is an important public health program aimed to screen all newborns. The role of the programme is secondary prevention aimed to provide early diagnosis and to prevent or ameliorate the long-term consequences of the disease for newborns who are suffering from Inherited Metabolic Disorders (IMDs).

The introduction of tandem mass spectrometry (MS/MS) in NBS offered the possibility of screening for almost 50 conditions using a single dried blood spot (DBS).

In Italy a nationwide newborn screening program for inborn errors of metabolism was institutionalized by law between 2016 and 2017 (Law 167/2016, https://www.gazzettaufficiale.it/eli/id/2016/08/31/16G00180/sg; DM 13 October 2016, https://www.gazzettaufficiale.it/eli/id/ 2016/11/15/16A08059/sg,; DPCM 12-1-2017, https://www. gazzettaufficiale.it/eli/id/2017/03/18/17A02015/sg).

Since 2016, Italy has thus made extended neonatal screening mandatory throughout the country for over 40 health conditions, resulting the top leader in Europe. They are divided into four main categories: aminoacidemias, organic acidemias, urea cycle defects and fatty acid oxidation disorders.

Through advances of genetic testing technologies, it is nowadays also possible to use DNA sequencing from DBS to test for genetic diseases (genetic newborn screening, gNBS). First national screening programs have already integrated genetic testing in newborn screening to detect diseases like cystic fibrosis or spinal muscular atrophy. In parallel, the number of diseases, for which treatments are available are constantly increasing.

In Italy, the “Piano Nazionale Malattie Rare (2023-2026) (document attached) is a central “planning tool” in the field of rare disease. It is extremely relevant that this document has identified the following among the actions to be undertaken in the field of neonatal screening: “promote the collection of residual biological material from internal neonatal screening biobanks for use for diagnostic and research purposes”.

This study aims to explore the use of more extensive genetic testing to screen newborns for treatable genetic diseases.

In particular, gNBS is one of the pillars and the “core” of WP3 of S4C project; indeed, this work package (WP3) aims at exploring the use of gNBS in newborn as a gateway to shorten the diagnostic path and offer the opportunity for early treatment.

gNBS will adopt different strategies interrogating currently treatable RDs (TREAT-panel-approach); Whole genome sequencing (WGS) of the entire Mendeliome (the list periodically updated of the pathological hereditary conditions) will be offered to symptomatic patients to identify known gNBS-escaped RDs and novel genes/phenotypes. WGS study is described as part of separated, specific ethical applications.
The TREAT panel is the tool which will be used in the current study and will include 244 disease genes that are related to treatable genetic disorders, prioritizing those with early onset and where natural history key elements are known. Other 7 genes are also included in the TREAT-panel. These genes are not disease genes but are technical internal controls we will screen especially to identify copy number variations (namely deletions or duplications) in TREAT genes.
Genes were included in the TREAT-panel only when they fulfilled the following criteria: availability of an early, paediatric treatment, which is typically initiated during the first two years of life, disease onset during childhood, high penetrance, and known genotype-phenotype correlation.The gene/disease selection was a crucial step in gNBS planning and TREAT-panel design, therefore robust criteria and scoring have been applied to design the gene panels. Criteria for disease selection and list of TREAT genes, are listed in **Appendix II**.

Further, a sub-study (involving an additional cohort of 200 newborns, called TREAT-WGS cohort) is planned in Italy and is based on the application of WGS as a "technical validation method" on the TREAT-panel, meaning that exclusively the 244 genes included in the TREAT panel will be interrogated (TREAT-WGS). This TREAT-WGS validation study aims at testing the accuracy and the detection rate of the TREAT panel in the identification of both small variations and copy number variations. In this study the genetic output will remain those of the TREAT panel study, therefore only the 244 genes included in this panel will be interrogated "in silico" in this newborn cohort (TREAT-panel "in silico" on WGS backbone) for both small and copy number variations. No other genetic data will be provided to the couples and all materials already presented in the TREAT panel study application will remain unchanged. A new informed consent is needed, since the newborn screening method will be WGS and not the TREAT panel.
The same information, dissemination, and communicating tools of the TREAT-panel will be adopted: therefore, WGS will be carried out with the sole purpose of validating the technical "accuracy and sensitivity" of the targeted gene panel, allowing a comparison of feasibility, detection rate, and gene coverage (targeted TREAT-panel vs TREAT-panel *in silico* on WGS backbone) in detecting small and copy number variations.
The application of WGS as a "technical validation tool" of the TREAT-panel will provide robustness to the TREAT panel diagnostic grade, for future application and translation into the health system for gNBS, especially for some variants, as copy number variations or structural variants, which may escape the TREAT panel testing. None of the non-TREAT genes genomic output generated by the WGS approach will be disclosed or provided to the couples and no risk of communicating "incidental findings"and "secondary findings" will occur, thus being a substudy fully compliant with the ethical rule of Screen4Care project.

The choice of using targeted sequencing, gene panel and the application of WGS as a "technical validation tool" maximises the screening ability to enrich for interpretable variation, including copy number variations and complex genotypes due to structural variants , provides a more understandable expectation to parents, allows rapid data processing and cost-effective data storage, and avoids many of the complex ethical issues associated with incidental findings and participant privacy inherent in whole exome or whole genome sequencing, being fully adherent to the World Health Organization ethical guidelines for genetic testing (https://apps.who.int/iris/handle/10665/63910, 1997). However, when patients present with early symptomatic disease, those who tested negative on the TREAT panel will be offered optional Whole Genome Sequencing (WGS) of the entire Mendeliome to recognize known gNBS-escaped RDs and novel genes/phenotypes.
In addition, communications with European Reference Networks (https://webgate.ec.europa.eu/ern/) will help map out post genetic report diagnosis work up and referrals and will ensure full access to positive infants to available therapies.

4 Study goals

Primary endpoints:

- Percentage of eligible couples who will accept to participate to the TREAT-panel (Cohort TREAT panel)
- Percentage of eligible couples who will accept to participate to the TREAT-panel in silico with WGS backbone (Cohort TREAT-WGS)
- Percentage of study participants in whom a pathogenic or likely pathogenic variant in appropriate inheritance model will be identified through TREAT-panel
- Percentage of study participants in whom a pathogenic or likely pathogenic variant in appropriate inheritance model will be identified through TREAT- WGS
- Percentage of study participants resulted fully negative at the gNBS (through both TREAT-panel and TREAT- WGS)

Secondary endpoints:

- Clinical and therapeutic follow-up of infants with positive findings in gNBS (through ERN collaboration and guidelines)
- Carrier frequency of recessive diseases (both autosomal and X-linked) and percentage of variants of unknown significance identified through gNBS (through both TREAT-panel and TREAT-WGS)
- Percentage of infants resulted positive at the gNBS TREAT-panel (through TREAT-panel and TREAT-WGS) who got access to the approved existing approved treatments
- Technical assessment of the TREAT-WGS
- Comparison of feasibility and detection rate (TREAT-panel vs TREAT-WGS)

- Comparison of gene coverage (TREAT-panel vs TREAT-WGS)
- Evaluation of number and type of reported variants (TREAT-panel vs TREAT WGS)
- Assessment of couples/expecting parents' compliance to a WGS screening test (assessed through parents' decision to accept/decline the TREAT-panel when WGS as testing method is proposed)

Please note: All infants with positive findings at gNBS (through both TREAT-panel and TREAT-WGS) will be addressed into the standard of care procedures at the ERN excellence center for diagnosis and treatment. **Appendix III** shows the list of ERNs in which University Hospital of Ferrara and OPBG (Italian S4C centers) are Health Care Providers and representatives.

5 Study population

5.1 General

Participation in the study can be offered to all parents and/or legal guardians of a newborn at participating hospitals and related birth centers. It is planned to include 18.000 newborns. Only couples with signed consent are allowed to participate in the study. Both parents must sign the informed consent to get their infant enrolled in the TREAT-panel study or in the TREAT- WGS .

5.2 Inclusion and exclusion criteria

Inclusion criteria:

- Newborns born in one of the participating hospitals
- Informed consent signed by both parents/legal guardian to participate in TREAT-panel
- Informed consent signed by both parents/legal guardian to participate in TREAT- WGS

Exclusion criteria:

- Lack of the above criteria

5.3 Recruiting

Recruitment of research participants will be performed using the process already adopted for the metabolic newborn screening programs in participating countries. In Italy, this includes 48 rare diseases and is well established in all Regions, although with some differences. Following the already existing NBS pipeline will ensure to address and recruit a representative sample without neglecting any minority meeting the concepts of equality and equity also in relation to the well-known heterogenous ethnic

composition of European population due to the important immigration phenomenon which is ongoing in all Member States.
The recruitment will be competitive and will last at least 12 months (with the possibility of extension to reach the enrolment target).
In detail, the information to the parents will be carried out through dedicated videos, flyers/leaflets, and meetings during one or more pregnancy checkpoints (CKPTs): an early CKPT in the first half of pregnancy, a major checkpoint at the end of pregnancy and a rescue checkpoint just after birth at DBS collection for biochemical NBS.
The administration of dedicated videos at the various CKPTs will take place always in the presence of healthcare personnel (gynaecologist/midwife/neonatologist/geneticist). The dedicated videos will also be available online -S4C website (https://screen4care.eu/)- should the couple wish to review them later; moreover, it will be possible to make requests for further clarification since the dedicated project' email address trialsgeneticamedica@unife.it is available. Furthermore, on the S4C website will be published a poster describing the procedure and a section dedicated to FAQs ("*Frequently asked questions*") is also planned.
If parents give consent to project' participation, a project dedicated DBS card reporting the S4C logo (S4C-DBS) will distributed at S4C birth centers to collect blood samples from enrolled neonates and then shipped to the S4C sequencing Hub (OPBG, Rome Italy) where all European samples, will be sequenced.

Important Note:

The couples' choice to participate to the two different cohorts' studies (TREAT-panel and TREAT-WGS) will be free, recruitment will be competitive in both cohorts, meaning that when reached the established number of infants to be screened (200 for TREAT-WGS, and 18.000 for the TREAT panel) the recruitment will be closed for both cohorts.

A schematic representation of gNBS pipeline is reported in **Appendix IV.**

6 Methods and realisation

6.1 Sample collection and analysis

Samples will be collected on the dedicated S4C DBS card in parallel with the collection of the DBS card of the metabolic newborn screening. Then samples will be pseudonymized at S4C birth centres and shipped to OPBG Hub, for DNA extraction and TREAT-panel NGS sequencing.
For a list of genes screened by panel for TREATable diseases see **Appendix V**.
Extracted DNA from S4C DBS cards will be conserved as pseudonymized at the OPBG hospital for the entire S4C duration, as described in Deliverables. OPBG will be responsible partner for sample conservation. If specified and agreed in the informed consent, S4C DNAs can be used for research purpose only for future S4C activities only, in their strictly anonymized habit. In some (expected a few) cases of cohort TREAT-WGS, when DNA quality extracted from the DBS might not be adequate, a

newborn peripheral blood sample taken might be needed and requested to the couple, as clearly mentioned in the informed consent.
The OPBG Hub will transmit by a dedicated, encrypted cloud platform BaseSpace (the S4C data repository, described in WP2) the raw data of gNBS sequencing output. Data will be therefore analyzed by two different partners: CNAG (Centro Nacional de Analisis Genomico) in Barcelona and Genoox in Tel Aviv, which will have access to this platform and are fully committed to be adherent to the ethical and confidentiality rules of S4C, as described in Deliverable D6.11 (document attached).
Results will be available to consortium members and through the RD-Connect GPAP and Genoox/CNAG platforms (whose access is limited to S4C consortium partners only and double-checked). These will make the data FAIR-field and sharable within the project in a privacy-preserving and controlled data access environment. As the aim of the TREAT-panel is to identify diseases where early diagnosis and treatment might benefit the health of the affected infant, the scientific report (prepared and finalized by CNAG and GENOOX) will report all variants identified in the TREAT genes. This report will be delivered to S4C validation Hubs (OPBG,UNIFE and Freiburg) that will perform variants' evaluation ("manual curation") and eventually a technically validate the identified variants.
In all newborns (both cohorts) in which the "manual curation" of the scientific report (i.e. variants' evaluation) and the eventual technical validation will confirm the pathological genotype, the couples will be contacted by birth centers who will direct them to the closest (disease specificity and logistical point of view) competent ERNs.
In detail, to be compliant with the Consortium Agreement rules, the birth centers will provide the family with a written curated scientific report (a document intended exclusively for research purposes) including the closer and competent ERN address and contacts.

The ERN centre will provide a diagnostic grade report (requiring a diagnostic confirmation performed on a new sample from the newborn and on the parents).The diagnostic report will include only the following genetic findings:

- Pathogenic or likely pathogenic variants in appropriate inheritance model and according to penetrance characteristics

VUS will not be validated nor reported in the scientific and in the diagnostic report. In case of identification of a VUS, a pipeline to further study *in silico* or by functional studies of the VUS will be established at the ERN clinical and diagnostic centres as part of the research flow also (if feasible) via collaboration with the European Joint Programme ERDERA (Preparing the path to the European Rare Diseases Research Alliance) which will be dedicated to VUS collection and analysis. As already mentioned above, only TREAT panel-related genes variants will be called and reported.

Summarizing, a scientific report will be generated by CNAG/GENOOX, and this will come back to S4C Hub (OPBG UNIFE, and Freiburg) that will provide "manual curation" (i.e. variants' evaluation) and eventually technical validation of the identified variants.

The birth centers will provide the family with a written curated scientific report (a document intended exclusively for research purposes) including the closer and competent ERN address and contacts.
The ERN centre will be in charge to provide a diagnostic grade report, which requires a diagnostic confirmation performed on a new sample from the newborn and on the parents.
We estimate that more than >95% of study participants will not carry any of the above-mentioned pathogenic or likely pathogenic variants as for rare or ultrarare diseases included in the TREAT panel.

The gNBS (through TREAT-panel and TREAT-panel in silico with WGS backbone) may therefore have three results:

- a negative result: in this case the family will receive a written information (via "PEC" mail or by digital tools, including electronic health records –EHRs- or -subject to the approval of the DPO- "*fascicolo sanitario*" in Italy) stating that the S4C did not identify any variant in the TREAT disease genes
- result with variants of unknown significance: in a case of an identification of a VUS, a pipeline to further study *in silico* or by functional studies of the VUS will be established at the ERN clinical and diagnostic centre (if applicable through the ERDERA project). No result will necessarily have to be communicated to the couple.
- a positive result: in this case the family will be contacted by the Birth Center which will provide a written curated scientific report and then will address the couple and baby to ERNs for a multidisciplinary post-test counselling for diagnosis communication, explanation of clinical and reproductive implications, access to available therapies and clinical and instrumental follow-up
- Carrier status for recessive pathogenic variant will be reported only if agreed on the informed consent, and provided on separate form. Since positive babies data analysis and communication is a priority of S4C, carrier status will be eventually communicated as a secondary endpoint and only in Italy, at the end of the project, and when all positive cases would have been solved and correctly addressed to ERNs.

As above mentioned after completion of the study all data will stored at the S4C repository (WP2) and can be used for additional research purposes only and fully anonymized. To do so, the results will only be reported as aggregated data to avoid potential re-identification of study participants.

Regarding the estimation of critical cases identifiable through TREAT-panel, if we consider the number of total newborns enrolled in S4C study (approximately 18.000 for the cohort TREAT-panel and 200 for the cohort TREAT-WGS)), we therefore expect to identify approximately 500 newborns in Europe (Italy, France, Germany, Czeck Republic, Greece, Poland), approximately 250 newborns in Italy and approximately 125 newborns in Emilia-Romagna.

Appendix VI shows a Figure with estimation of critical cases identifiable by TREAT-panel based on the frequency of rare, very rare and ultrarare diseases (as per Eurordis study, Reference reported in the Figure).

6.2 Follow-up for study participants

Participants with no pathogenic or likely pathogenic variant

Negative result (expected to be about >95% of screened infants) will be communicated to the couple by written information (via "PEC" mail or by digital tools, including electronic health records –EHRs- or -subject to the approval of the DPO- "*fascicolo sanitario*" in Italy) stating that the S4C did not identify any variant in the TREAT disease genes. If participants should develop symptoms suggestive of a genetic disease within 12-24 months of life, whole genome sequencing of the entire Mendeliome will be offered for further clarification (not described here, since part of the separate specific ethical application).

Participants with pathogenic or likely pathogenic variants

As anticipated S4C validation Hubs (OPBG, UNIFE, and Freiburg) will provide "manual curation" (i.e. variants' evaluation) and eventually technical validation of the identified pathogenic/likely pathogenic variants with appropriate inheritance model and according to penetrance characteristics. If validated, the birth centers will provide the family with a written curated scientific report (a document intended exclusively for research purposes) including the closer and competent ERN address and contacts. All S4C partners involved in gNBS are members of many ERNs (see the list in the **Appendix III**) therefore the path to address positive infants to excellence diagnostic centers will be timely and smoothly planned. All phenomic categories associated with disease-genes included in TREAT-panel will be therefore followed up by the relevant reference ERN (**Appendix VII**).

7 Assessment of benefits and risks

7.1 Benefits of participation

Study participants might benefit from early diagnosis of a rare genetic disease and consequent timely access to treatments, as per the definition of the TREAT-panel, where genes were primarily scored for the availability of treatments in the corresponding rare diseases.

The criteria of treatability implies ''Approved drug treatment (EMA) that includes gene therapy and/or other treatment/intervention (drug, diet, bone marrow transplantation, supplements, vitamins etc) that is recommended by guidelines (at least for a subgroup of the disease) and treatment available in Italy'' (see **Appendix II**).

It is well known that benefit might be greater if treatment is initiated in the pre-symptomatic or early stage of the disease. Early diagnosis of a rare disease might also reduce the burden of multiple diagnostic tests and periods of uncertainty that are often associated with the "diagnostic odyssey" of a rare disease. Study participants with no abnormalities might benefit from the reassurance that presence of any of the rare diseases screened is very unlikely (accuracy grade 97%).

Regarding the application of WGS as a "technical validation tool" of the TREAT-panel, this approach can result in a number of benefits.

Indeed, since exclusively the genes included in the TREAT-panel will be interrogated, this innovative approach minimises the amount of data generated and the presence of VUS and lacks the risk of communicating "incidental findings" or "secondary findings"; moreover, it offers the great potential to technically validate the TREAT panel output especially from some variant types as copy number and structural variants. The eventually stored genomic data might be reconsidered fo further research use, pending the signing of the informed consent.

7.2 Risks of participation

The blood samples for the genetic newborn screening are taken in parallel with the national metabolic newborn screening (both on DBS cards). Thus, there are some procedure-related risks, i.e. flushing or pain for the participating infant and a small risk of infection, but the midwife will wear gloves and clean the heel of the babies before collecting the blood to reduce the risk as far as possible. Regarding the genetic analysis carried out, it is possible that the test performed results negative because of the limitation of the technique used (for this reason, the quality parameters of the analysis are indicated in the report). In addition, parts of some genes may have characteristics that make their analysis problematic: such parts may be partially excluded from the results because they are difficult to evaluate by sequencing techniques. In light of this, the sub-study based on WGS as a technical validation tool, is planned to compare the feasibility, detection rate and gene coverage of the two methods (TREAT-panel vs TREAT-panel in silico with WGS backbone) thus conferring robustness to the TREAT panel diagnostic grade..

Finally, the gNBS may also identify carriers of recessive (both autosomal and X-linked) diseases, although this is not the goal of the gNBS protocol. Carrier status might be communicated if agreed in the informed consent (only upon request by legitimate subjects, i.e., parents or legal tutors).

a. Medical defensiveness

All participating countries have an established national newborn screening program, which tests for a variable number of metabolic markers (depending on the country) and in some countries also specific genetic markers. Participation to the established national newborn screening is mandatory for study participants (birth centers) to follow up the established national NBS pipeline. Since S4C is an EU funded research project and not a public health program, we cannot either interfering and possibly hampering the national metabolic NBS, nor grant a precise turnaround reporting time for diagnosis. High throughput and parallelism of infant samples and data are mandatory to be adherent to the available budget, to screen 18.000 babies, and to grant large outputs to be analysed. It is possible, therefore, that a very few infants may have already developed symptoms when the diagnostic report become available.

Nevertheless, this might occur only for those genetic diseases not covered by the national metabolic screening (in Italy the SNE), and which are only screened using the TREAT panel, meaning in a very limited number of babies. However, since S4C aims at maximizing translatability of gNBS into health system we commit to provide the diagnostic grade report as soon as possible and approximately by 120 days from the DBS collection as it is stated in the informative form. Participation to the S4C research project will not therefore have any no impact on the routine care and on the SNE and will provide additional benefit as early diagnosis and early access to treatments for diseases not currently screened by national NBS-SNE. It has to be noted that treatment availability might be slightly different depending on the Member State. In these cases, treatment access will be facilitated also via S4C consortium contacts and ERNs, and all infants will always receive standard of care in the routine day life.

8 Biometrics

Statistical analysis of the data will be mainly descriptive and report frequency of different variants in the study samples. Ethnic differences will be also analysed as country specific differences concerning the acceptance of gNBS and impact on participating families will be evaluated.
Although RD incidence estimation is not the primary scope of S4C, frequency of pathogenic and likely pathogenic variants will allow relevant epidemiological observations.

9 Ethical and legal aspects

9.1 Person responsible for data management and data collection

The S4C scientific coordinator and the WP3 project leaders are the primary persons responsible for the quality of data collection. The Scientific Coordinator is fully responsible for the data management as well as the storage of the data. For each study birth/clinical centre a responsible person has been already nominated. The Code of Ethics of S4C is attached (D1.6).

9.2 Information of participants and informed consent

Parents/legal guardians will receive detailed information on the nature, objectives, possible risks and benefits of the research. Dedicated videos (in Italian and English) will be prepared to illustrate the S4C project to the couple at the different check points. A leaflet will be also prepared and a poster describing the procedure will be also loaded on the S4C website. Participants will be given enough time to consider their participation and ask researchers questions about the research. They will further be informed that at any time they can drop down their participation and revoke the informed consent.
Participants have the right to be informed on the data collected and to correct any mistakes in personnel data. Participants can only take part in the study if an informed consent form is signed. The investigators

will store the original (both paper or digital) signed informed consent at the birth/clinical sites place and corresponding ITC department.

9.3 Pseudonymisation

Due to the direct interaction of the researchers with the participants, it is impossible to collect the data anonymously. The obtained information will be barcoded to allow confidential and anonymous processing and reporting of the data. The identity of the participants will not be shared with all S4C partners so the privacy will be guaranteed.

All participant-related data will be coded in such a way that individual persons cannot be identified from the corresponding data (directive 95/46/EC) according to regulations (unique pseudocode identifier where possible). Identities will be pseudonymised at S4C birth/clinical centers, where the informed consent is collected, data/biomaterial will be barcoded with the pseudonymised identifiers and a separate database linking the unique pseudocode to the participant's names will be used. This database with names will only be available to the birth/clinical center PI. No personal data will be stored or be accessible off-site.

The research data can be used for publications in scientific journals and writing of reports or publication for the S4C project. The study results can be published anonymously, whereby it will not be possible to draw conclusions about the identity of the participating persons.

9.4 Data collection

Data collection and data management will be conducted in compliance with the principles of the Declaration of Helsinki (1996), with the principles of Good Clinical Practice (GCP), with the GDPR (General Data Protection Regulation) as well as with national data protection regulation of participating countries.

For the participation in the study the following data are recorded: gender, name, date of birth, blood sample, inclusion and exclusion criteria.

Possible disease-relevant data such as gender, age, phenotypic characteristics and date of examination, as well as diagnosis of genetic analysis, confirmation of diagnosis and therapy will be collected and shared in the database.

All raw data, such as patient files, are source documents. Their availability is ensured for routine monitoring. The participation of the individual participant in the study is documented. The study leader maintains an independent list for the identification of the study participant. This list contains the names and date of birth as well as pseudonymisation codes of the participants. The study leader is responsible for the quality of data collection and storage. All data will be collected in the following repositories:

- Birth/clinical centers Hospital certified (ITC) repositories
- CNAG and GENOOX datasets
- S4C cloud

9.5 Data transfer and data analysis

Prior to a scientific analysis of the materials and data of this study, all information will be pseudonymised according to the guidelines of the Federal Data Protection Act. Pseudonymised blood samples for NGS are sent to OPBG Hub. The completed sheets are kept in the data collection form in the study centre. The study leader of each study centre maintains an independent list for the identification of the study participants.

The pseudonymised data can be shared within the members of the consortium. The code, being the identification numbers linked to the identity of the participants will be held confidentially within the group of involved researchers of the respective study birth/clinical centre. Only the researchers that are involved in this study will have access to this code and only they will know the identity of participating stakeholder members.

The data is transferred to the database for subsequent computer-aided data analysis as well as for collaborative match making and interpretation of genetic variants.

The data will only be passed on pseudonymously on encrypted physical drives. The data will not be used for later approval of the prototype used. S4C involves the collection, use and sharing of personal sensitive data (GDPR Art.2 No.2) among the different partners, implying that personal data will be transferred also between partners and across national borders within Europe and in EU affiliated country partner (Genoox, Tel Aviv, Israel). All personal data generated and processed in S4C will be protected according to generally accepted principles of information security. A personalized identity and access management system will be implemented, ensuring that access to personal data will only be granted to authorised personnel. All personal data flows will be possible only via secured and encrypted channels. Passwords or encryption codes will be always communicated separately from the information sent. Data management and sharing is described in the attached D6.11.

9.6 Data protection

The involved researchers commit to the highest standards of data security and protection in order to preserve the personal rights and interests of study participants.

The involved researchers will follow the consents as given by the parents/legal guardian, the principles of the new European General Data Protection Regulation (directive 2016/679 (http://eur-lex.europa.eu/legal-content/EN/TXT/?uri=CELEX:32016R0679) on the protection of individuals with regard to the processing of personal data and on the free movement of such data as implemented into national law and in general the principles laid out in the GA4GH "Framework for Responsible Sharing of Genomic and Health-Related Data" (https://genomicsandhealth.org/framework).

Data Protection Officers (DPOs) appointed at each birth/clinical centers will receive this protocol together its eventual approval to monitor the privacy aspects of the S4C study.

9.7 Data storage

All downstream research data is stored on secure servers accessible only to authorized users within the participating organisations. Genomic data is considered unique but does not identify a participant on its own without combination with other data. Nevertheless, all genomic datasets are stored on secure servers accessible only to authorized users within the participating organisations separated from the personal data.

Identifiable participant personal data will be stored in secure records at the S4C clinical site. This institution will assign a non-identifiable alphanumeric code to the participant and store the key in a secure repository. Both data storage including regular backups and data security will be managed by the clinical site in line with their existing best clinical practice and appropriate national data protection regulations for identifiable clinical data.

As email addresses might contain identifiable personal data, we will use an independent data trustee to store pseudonymisation codes and corresponding email addresses. Through this data trustee, it will be possible to distribute online surveys to study participants by email.

Genomic data submitted to S4C is stored during analysis on high performance servers at Genoox and CNAG disconnected from the general Internet and with appropriate user permissions. Processed genomic data is indexed in a non-relational database within a server with limited internet access. Processed data is accessed through an API accessible only to authorized users logging in to a Central Authentication System. All data is backed up regularly.

There will be two cohorts:

1) Approx. >95% of screened infants will be without pathological findings. For these infants there is only little data stored: Name, pseudonymisation code, without pathological findings.

2) Regarding screened infants with pathological findings suggestive of a genetic disease, data stored for these infants will include name, pseudonymisation code, diagnosis, symptoms, therapy and confirmation of diagnosis.

The TREAT panel/TREAT-WGS data will be conserved and stored at the S4C platforms and at the birth/clinical centers, and in the S4C cloud for the S4C entire duration and then they can be made available for research purposes only as aggregated data sources, if this has been explicitly agreed in the informed consent.

The deliverable D6.13 (annex) reports all data storage and NBS studies pipelines in more detail and is an extensively revised update of D6.12.

10 Appendices:

10.1 Appendix IA: General Screen4Care project overview

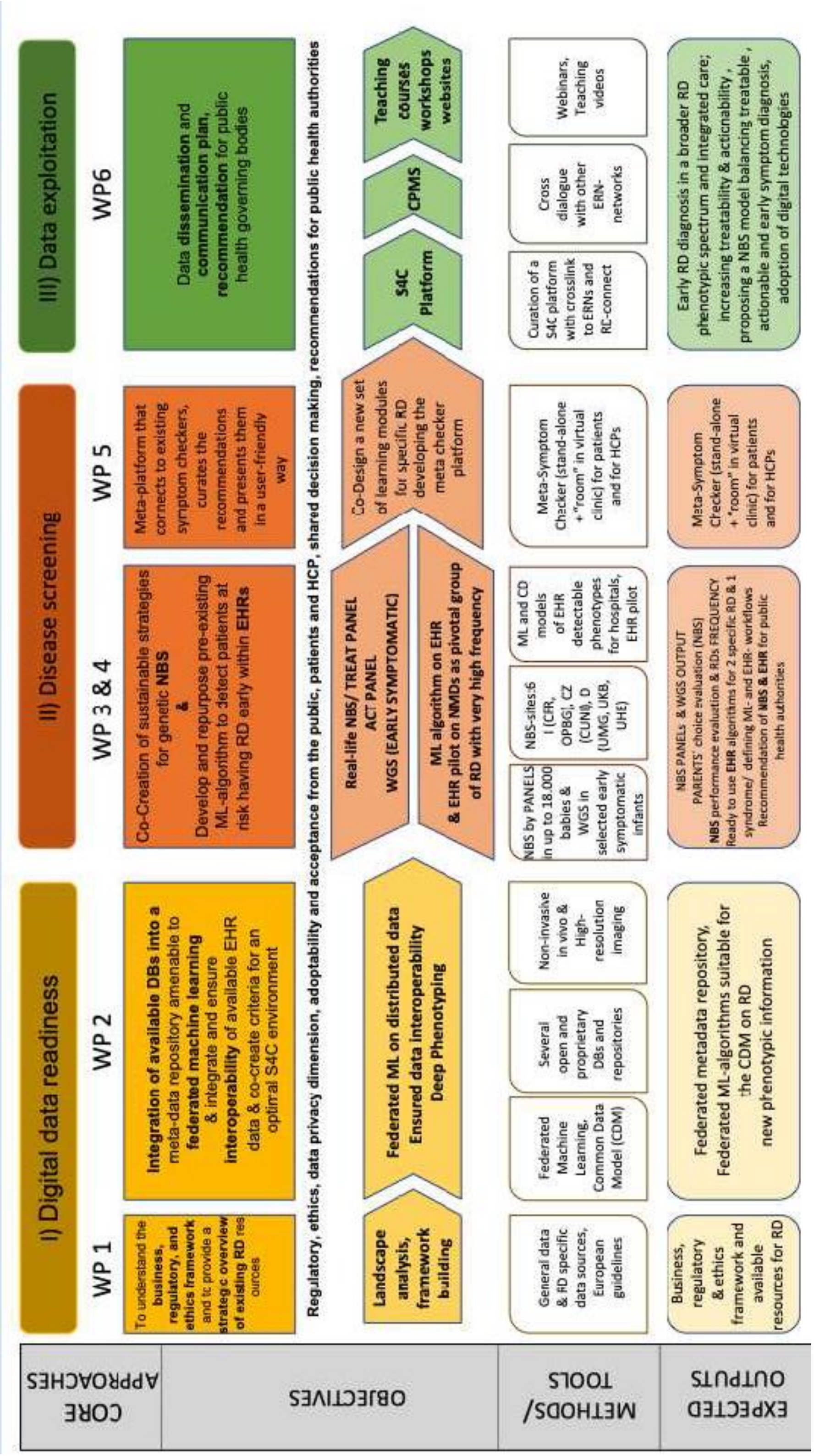

Appendix IB: Schematic representation of S4C structure organized into six interdisciplinary areas of focus

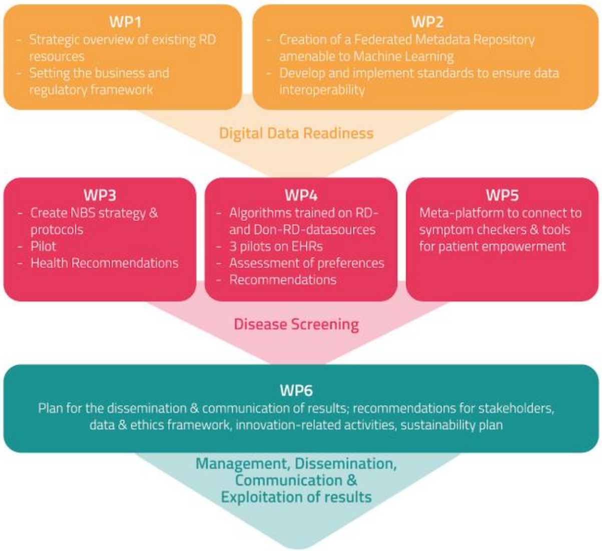

10.2 Appendix II: Criteria to select diseases for TREAT-panel

CONFIDENTIAL

1) TREATability

scoring	Definition
YES	Approved drug treatment (EMA) incl. gene therapy and/or other treatment/intervention (drug, diet, bone marrow transplantation, supplements, vitamins, etc) that is recommended by guidelines (at least for a subgroup of the disease) **and**<br>Treatment available in Germany and Italy
NO	All genes/diseases not fulfilling the criteria above

2) Disease onset

Scoring	Definition
2	Predominantly paediatric onset of disease
1	Spectrum of onset across age groups, difficult to predict onset/ limited knowledge about natural history of disease
0=No	Mainly adult onset (> 18 years)

3) Disease severity

Scoring	Definition
2	Most likely to cause significant health problem
1	Spectrum of severity, difficult to predict
0=No	Not causing significant health problem

4) Penetrance

Scoring	Definition
2	Penetrance > 80%
1	Intermediate penetrance (20-80%)
0=No	Low penetrance (< 20%)

5) Clinical validity

Scoring	Definition
2	known pathogenic variants with clear phenotype-genotype correlation
1	genes with known pathogenic variants and partial genotype-phenotype correlation (as in ultrarare conditions)
0=No	genes with only benign variants or variants of unknown significance, no established genotype-phenotype correlation

1

10.3 Appendix III: List of ERNs in which University Hospital of Ferrara, OPBG participate

The list of ERNs in which OPBG participates

1. **BOND**
2. **EUROBLOOD**
3. **ERNICA**
4. **ERN RARE-LIVER**
5. **ERN GENTURIS**
6. **GUARD HEART**
7. **EYE**
8. **METABERN**
9. **RITA**
10. **ITHACA**
11. **VASCERN**
12. **EPICARE**
13. **RND**
14. **EURO-NMD**
15. **PAED CAN**
16. **ERN LUNG**
17. **ERKNET**
18. **ERN SKIN**
19. **EUROGEN**
20. **ERN TRANSPLANT CHILD**

The list of ERNs in which University Hospital of Ferrara participates

1. **Endo-ERN**
2. **ERN ITHACA**
3. **ERN EuroBloodNet**
4. **ERN Euro-NMD**

10.4 Appendix IV: Schematic representation of gNBS pipeline

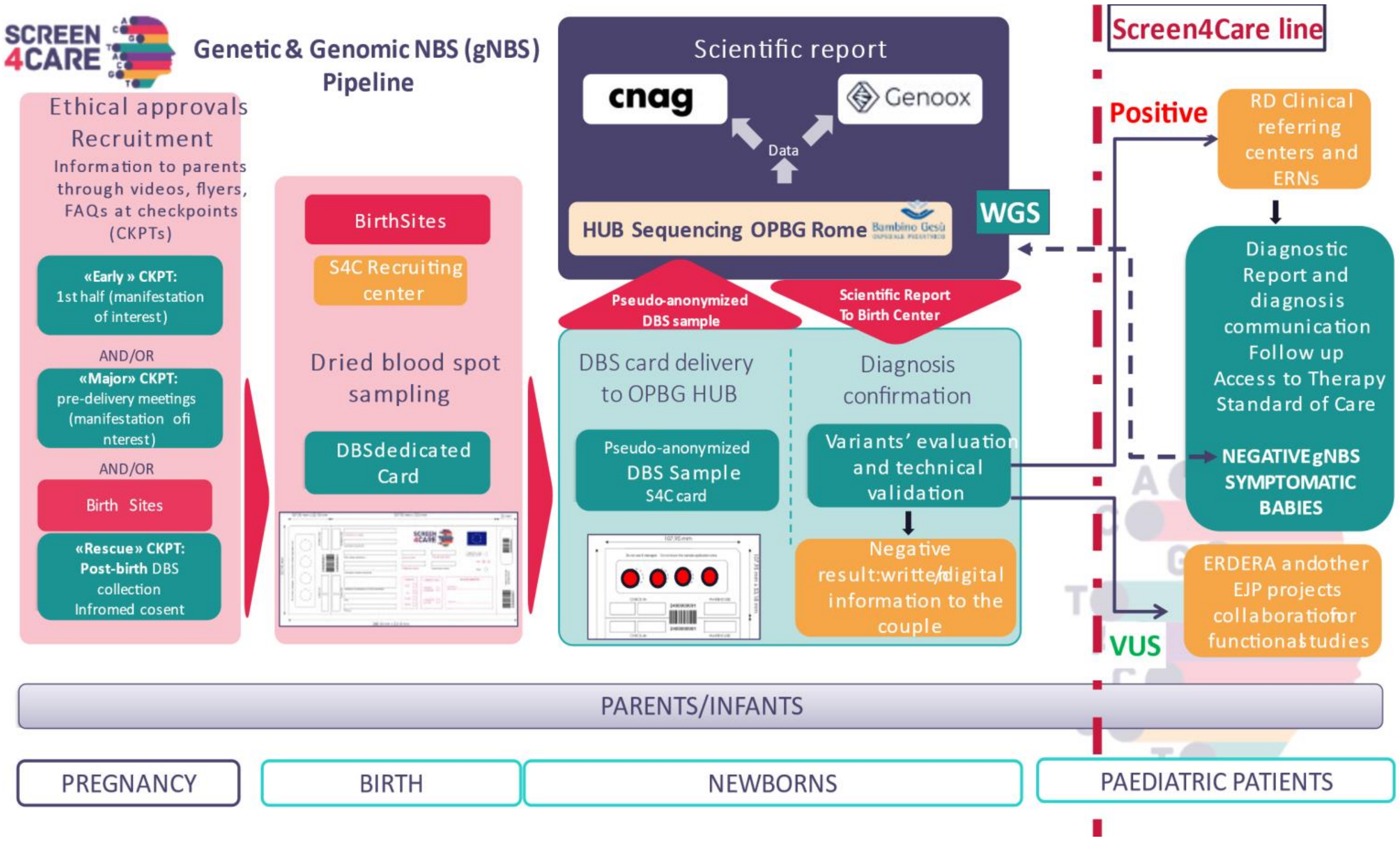

10.5 Appendix V: Reference genes technical table and list of 244 genes included in the TREAT- panel

Symbol	Gene-MIM
ABCC8	600509
ABCD4	603214
ACAD8	604773
ACADM	607008
ACADVL	609575
ACAT1	607809
ACVR1	102576
ADA	608958
ADA2	607575
ADAMTS13	604134
AGL	610860
AGRN	103320
AGXT	604285
ALDH7A1	107323
ALDOB	612724
ALG14	607905
ALG2	612866
ALPL	171760
ARG1	608313
ARSA	607574
ARSB	611542
ASL	608310
ASS1	603470
ATP6V0A4	605239
ATP6V1B1	192132
ATP7A	300011
ATP7B	606882
AUH	600529

BCKDHA	608348
BCKDHB	248611
BCKDK	614901
BTD	609019
BTK	300300
CA2	611492
CACNA1C	114205
CBS	613381
CD40LG	300386
CFTR	602421
CHAT	118490
CHRNA1	100690
CHRNB1	100710
CHRND	100720
CHRNE	100725
COL13A1	120350
COL1A1	120150
COL1A2	120350
COLQ	603033
COQ2	609825
COQ6	614647
COQ8B	615567
CPS1	608307
CPT1A	600528
CPT2	600650
CTNS	606272
CYP11A1	118485

CYP11B1	610613
CYP27A1	606530
CYP27B1	609506
DBT	248610
DCLRE1C	605988
DDC	107930
DMD	300377
DOCK8	611432
DOK7	610285
DPAGT1	191350
DUOX2	606759
DUOXA2	612772
ELANE	130130
ETFA	608053
ETFB	130410
ETFDH	231675
F13A1	134570
F2	176930
F7	613878
F8	300841
F9	300746
FAH	613871
FANCA	607139
FANCB	300515
FANCC	613899
FANCD2	613984
FANCE	613976
FANCF	613897
FANCG	602956
FANCI	611360
FANCL	608111
FERMT3	607901
FGA	134820
FGB	134830
FGFR3	134934
FGG	134850
FOXN1	600838

FOXP3	300292
G6PC1	613742
G6PD	305900
GAA	606800
GALC	606890
GALK1	604313
GALNS	612222
GALT	606999
GAMT	601240
GATA1	305371
GATM	602360
GBA1	606463
GCDH	608801
GCH1	600225
GCK	138079
GFPT1	138292
GLUD1	138130
GMPPB	615320
GNAS	139320
GUSB	611499
HADH	601609
HADHA	600890
HADHB	143450
HBA1	141800
HBA2	141850
HBB	141900
HLCS	609018
HMGCL	613898
HPD	609695
IDS	300823
IDUA	252800
IGF1	147440
IGSF1	300137

IL2RG	308380
IL7R	146661
INS	176730
ITGB2	600065
IVD	607036
JAK3	600173
KCNH2	152427
KCNJ11	600937
KCNQ1	607542
LHX3	600577
LIPA	613497
LMBRD1	612625
LRP4	604270
LYST	606897
MAN2B1	609458
MLYCD	606761
MMAA	607481
MMAB	607568
MMACHC	609831
MMADHC	611935
MMUT	609058
MPI	154550
MTHFR	607093
MTR	156570
MTRR	602568
MUSK	601296
MVK	251170
NAGS	608300
NBN	602667
NHEJ1	611290
NKX2-1	600635
OTC	300461
PAH	612349
PCCA	232000
PCCB	232050
PDSS2	610564

PHEX	300550
PHKA2	300798
PHKB	172490
PHKG2	172471
PIK3CD	602839
PIK3R1	171833
PKLR	609712
PLPBP	604436
PMM2	601785
PNPO	603287
POU1F1	173110
PRF1	170280
PROP1	601538
PSAT1	610936
PTS	612719
QDPR	612676
RAB27A	603868
RAG1	179615
RAG2	179616
RAPSN	601592
RB1	614041
RET	164761
RPE65	180069
RPL11	604175
RPL35A	180468
RPL5	603634
RPS10	603632
RPS17	180472
RPS19	603474
RPS24	602412
RPS26	603701
RPS7	603658
SBDS	607444
SCN4A	603967
SCN5A	600163

SCNN1A	600228
SCNN1B	600760
SCNN1G	600761
SH2D1A	300490
SLC12A3	600968
SLC16A1	600682
SLC18A3	600336
SLC19A3	606152
SLC22A5	603377
SLC25A1	190315
SLC25A13	603859
SLC25A15	603861
SLC25A20	613698
SLC26A3	126650
SLC26A4	605646
SLC2A1	138140
SLC37A4	602671
SLC46A1	611672
SLC52A2	607882
SLC52A3	608761
SLC5A5	607882
SLC5A7	613350
SLC6A8	300036
SLC7A7	603593
SMN1	600354
SMPD1	607608
SPR	182125
STAR	600617
STX11	605014
STXBP2	601717
SYT2	600104
TAT	613018
TCIRG1	604592
TG	188450
TH	191290
THRA	190120
TK2	188250

TMEM70	612418
TPO	606765
TPP1	607998
TSHB	188540
TSHR	603372
UNC13D	608897
VAMP1	185880
VWF	613160
WAS	300392
XIAP	300079
ZAP70	176947

***further genes included in the design of *TREAT-panel* because of technical and design related issues**

GENE SYMBOL	NM or NR	CHROMOSOME	LOCUS
SMN2	NM_017411.4	5	q13.2
GUSBP14	NR_024054.2	5	q13.2
GTF2H2B	NR_033417.1	5	q13.2
GUSBP15	NR_034021.1	5	q13.2
GUSBP16	NR_146391.1	5	q13.2
NAIP	NM_004536.3	5	q13.2
SERF1A	NM_021967.4<br>NM_022968.2	5	q13.2

10.6 Appendix VII: Estimation of critical cases identifiable by TREAT-panel based on the frequency of rare, very rare and ultrarare diseases

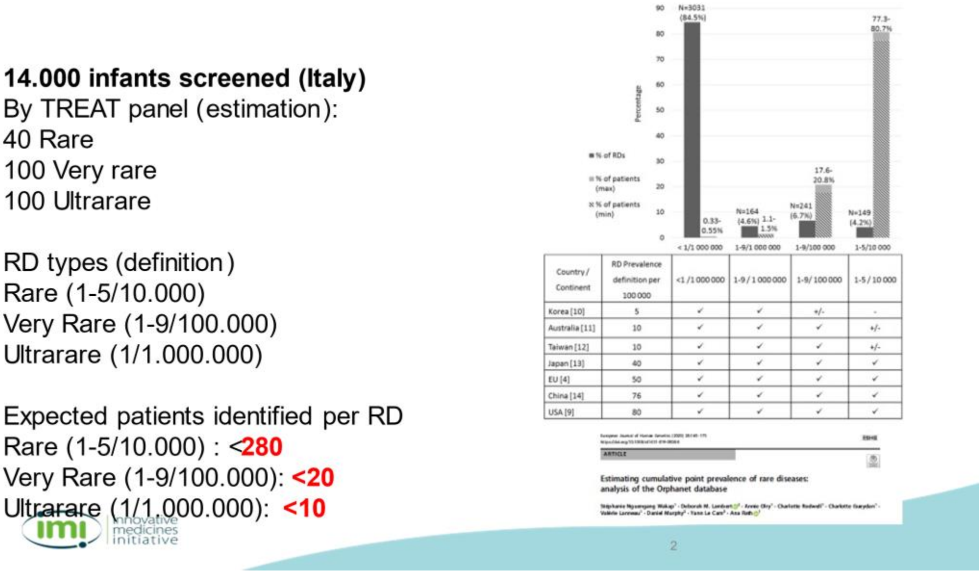

Country / Continent	RD Prevalence definition per 100 000	<1 / 1 000 000	1-9 / 1 000 000	1-9 / 100 000	1-5 / 10 000
Korea [10]	5	✓	✓	+/-	-
Australia [11]	10	✓	✓	✓	+/-
Taiwan [12]	10	✓	✓	✓	+/-
Japan [13]	40	✓	✓	✓	✓
EU [4]	50	✓	✓	✓	✓
China [14]	76	✓	✓	✓	✓
USA [9]	80	✓	✓	✓	✓

10.7 Appendix VII: Phenomic categories associated to disease-genes included in TREAT-panel and related ERN

Category	Total number of genes (out of 245) and associated ERN
Blood and coagulation disorders	33 - EuroBloodNet
Cardiological disorders	4 - GUARD-HEART
Endocrinological disorders	28 - Endo-ERN
Immunological disorders	26 - ERN RITA
Kidney diseases	9 - ERKNet
Metabolic (including mithocondrial disorders, oxidation disorders, lysosomial disorders, etc…)	106 - MetabERN
Neurolologic/neurodegenerative and neuromuscular disorders	25 - ERN-RND, EURO-NMD, ITHACA
Syndromic	6 - ITHACA
Others	7 - ERN-BOND, ERN-LUNG, ERN-EYE

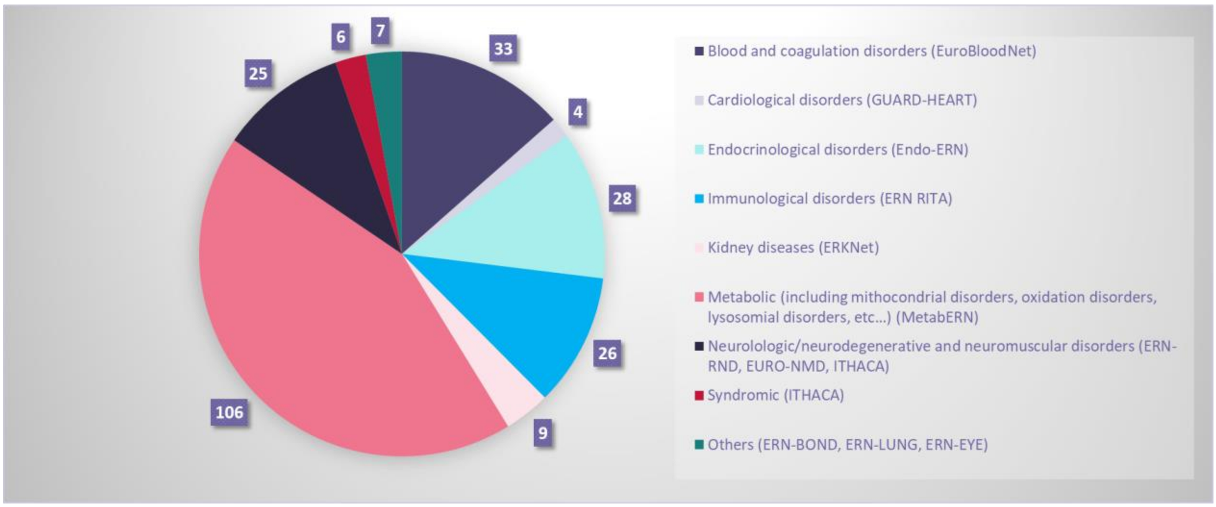

List of attached relevant documents

- Piano nazionale malattie rare 2023
- D6.11 Data management and Dissemination plan
- D1.6 Code of Ethics of S4C
- D6.2 Management guide
- D6.7 ELST Committee and Independent Ethics Advisor
- D6.1 Project management platform
- D6.19 Independent Ethical Advisor 1st report
- D6.12 Data Management and sharing plan
- D2.2 Viable implementation of the data repository
- D6.20 Independent Ethical Advisor 2nd report
- D6.13 Data Mangement and sharing plan (update of D6.12)
- List of TREAT-genes including all information related to treatability, OMIM, and other specific information
