# Supplementary material for: European Reference Networks as core health structures where referring genetic newborn screening positive infants: an innovative operational research framework
Source: Front Public Health. 2026 Jun 10;14:1822461. doi: 10.3389/fpubh.2026.1822461 (PMC13292599; doi:10.3389/fpubh.2026.1822461)
Supplement: Supplementary file 2 [file Data_Sheet_2.pdf]

SERVIZIO SANITARIO REGIONALE
EMILIA-ROMAGNA
Azienda Ospedaliero - Universitaria di Ferrara

Università degli Studi di Ferrara

DIPARTIMENTO INTERAZIENDALE MATERNO INFANTILE DIPARTIMENTO DI SCIENZE MEDICHE
U.O. DI GENETICA MEDICA
*Direttore: Prof.ssa Alessandra Ferlini*
https://www.ospfe.it/reparti/genetica-medica

***Consulenza Genetica***
***Genetica Clinica***
***Laboratorio di Genetica Medica***

**Informazioni**
Tel. 0532 974403-237773
*Via Fossato di Mortara, 74*
*44121 Ferrara*

**Prenotazioni**
*- In gravidanza: Tel. 0532 236491 (lun-ven, h 11-13)*
*- Altri casi: CUP Numero Verde 800532000*

*Sede Formativa della Scuola di Specializzazione*
***in Genetica Medica***
http://www.unife.it/medicina/geneticamedica

"Letterhead of the Institution"

# INFORMATIVE CONSENT FORM FOR PARENTS AND REPRESENTATIVES FOR PARTECIPATION IN EXPERIMENTAL STUDIES_TREAT PANEL

**Organization in which the study is carried out:** Unit of Medical Genetics, University Hospital of Ferrara

**Title of the study:**
Genetic newborn screening for treatable rare diseases through TREAT-panel within the Screen4Care project

**Project Lead:** Screen4Care (Scientific Coordinator: University of Ferrara)

**Local responsible of the study**: Prof.ssa Alessandra Ferlini

Dear Madam/ Sir,
in this center we intend to carry out a medical scientific research. A study is performed when physicians need to collect information about a problem.

To date, more than 7000 genetic diseases are known, diseases that affect less than one person every 2000.
These diseases, which overall involve 27-36 milion individuals in the EU and which affect one person in every 17 during their lifetime, are often severe,

multisystemic and chronic pathologies, which cause the risk of chronic, irreversible, and permanent diseases for patients.

Due to the nature of their conditions, rare disease patients and their families commonly experience delays in diagnosis, which can lead to serious consequences for their health and ability to plan their future.

The diagnostic delay also represents an obstacle to rapid intervention - such as effective treatment or enrollment in clinical trials- and to patient empowerment, which is achieved through various strategies, such as lifestyle changes, family planning, genetic counselling and coping with the psychosocial and/or economic consequences of own condition.

The Screen4Care research project aims at significantly reduce the time needed for the diagnosis and treatment of rare diseases through genetic newborn screening (gNBS) and Artificial Intelligence-based tools. The ultimate aim of the project is to improve the standard of care, access to new therapies and the quality of life of patients and their "*caregivers*".

For this study we would need your collaboration.

Participating in a study is an important decision.

Before you decide to accept or refuse to allow your child/beneficiary to participate in this study, please read this informative form carefully, taking all the time you need.

It is important that you ask us for clarification if something is not clear to you or you need further information.

Furthermore, if you wish, before deciding, you can ask your family members or a physician for advice.

If you decide not to participate in the study, your child/beneficiary will still receive all the expected assistance and physicians and other healthcare professionals will continue to follow him/her with attention. Your refusal to participate will not be interpreted as a lack of trust in them.

- **Why do we propose that your child/beneficiary participate in this study?**

We are suggesting that your child/beneficiary participate in this study because we intend to carry out a gNBS aimed at diagnosing some treatable rare genetic diseases before symptoms appear.

- **What does this study aim to do?**

This study aims to implement a gNBS, through the use of a multigene panel (called TREAT-panel) using the Next Generation Sequencing (NGS) approach, which will allow the analysis of approximately 245 genes associated with rare genetic diseases for which treatment is available.
This will allow us to act promptly and identify a targeted treatment to avoid the effects of some rare genetic diseases, before the symptoms appear.
In our project, treatment means “approved pharmacological treatment (EMA) that includes gene therapy and/or other treatment/intervention (drug, diet, bone marrow transplant, supplements, vitamins, etc.) recommended by the guidelines (at least for a subgroup of the disease) and that it is available in Italy".
The ultimate aim of the project is to improve the standard of care, access to new therapies and the quality of life of patients and their "*caregivers*".

*Specifically, the TREAT panel is a custom panel designed *ad hoc* in the S4C project and therefore, being a dedicated new synthesis product exclusively for research, cannot have an IVDR certification.

However, in the absence of IVDR certification, the sequencers and processes analytical of the TREAT panel will take place in accredited and certified laboratories, such as UNIFE and OPBG, which guarantees a monitoring system internal quality that will be implemented during the genetic screening with the TREAT panel.

The technical validation of the variants will instead be carried out at the laboratories belonging to ERN, therefore all accredited for diagnostics and with methods and reagents all marked IVDR, which will therefore guarantee a report with full diagnostic value (diagnostic grade).

- **Which genetic diseases will be investigated?**

The genetic diseases that will be analyzed in the TREAT-panel were selected on the basis of the availability of a treatment in Italy, which is generally started during the first two years of life; furthermore, rare genetic diseases that begin in early childhood and which affect only 1 child in 10.000 or even less were selected.

We will provide you with a list of genes that will be investigated (in paper and/or digital format).

- **What does participation in this study mean compared to the normal care your child/beneficiary receive?**

If you decide to have your child/beneficiary participate in the project, the study involves collection a few drops of blood taken from the heel at the birth center

for gNBS in parallel with that of the already known and mandatory extended neonatal screening.

For this purpose, a dried blood card (DBS) dedicated to the S4C project has been designed and will collected to the S4C center (UOC Medical Genetics, University Hospital of Ferrara); from here, the pseudoanonymized sample will be sent to the Medical Genetics Laboratory of the Bambino Gesù Pediatric Hospital in Rome where the DNA will be extracted from the DBS card and analyzed using TREAT-panel, to identify the treatable rare genetic diseases that we have selected for this study.

The sequencing data will be analyzed by two different analysis centers: CNAG (Centro Nacional de Analysis Genomico) in Barcelona, and Genoox in Tel Aviv (project partners committed to respecting S4C's ethical and confidentiality rules).

In most cases we do not expect to identify any genetic alteration: in this case you will receive written communication of the negativity of the analysis.

In the rare cases in which the genetic analyses carried out show some genetic alteration (pathogenic or probably pathogenic variants), we will contact you to discuss the results.

It may then be necessary to examine your child/beneficiary and perform further tests to confirm whether he/she actually has a genetic disorder.

If a genetic disorder is confirmed, we will also help you find an expert for that condition, so that your child/beneficiary can receive the best possible treatment and care.

Furthermore, if your child/beneficiary develops any health problems that may suggest a rare genetic disease within the first 12-24 months of life, the Screen4Care project is able to offer further genetic tests (such as whole genome sequencing, WGS).

In this case you will be asked to sign another informed consent form and an additional blood sample will be taken from your child/beneficiary.

**• What result can be obtained from this type of analysis?**

The analysis of the genes included in the TREAT-panel can lead to different results:

- negative outcome: no clinically significant variants have been identified based on current knowledge. In this case you will receive a written communication.
- variants of uncertain significance (VUS): variants in one or more analyzed genes that cannot be classified with certainty as either causative or benign, based on current knowledge. In this case, in-depth research assessments could be carried out, but it is not possible to guarantee a diagnostic report.
- positive outcome: pathogenic or probably pathogenic variants in disease genes associated with treatable genetic diseases. In this case you will be contacted and referred to an expert center for multidisciplinary post-test counseling, communication of the diagnosis, explanation of the clinical and reproductive implications, access to available therapies and clinical and instrumental follow-up.

The timing of communication of the results will be approximately 120 days from the time of collection of DBS (dry blood spot).

- **What are the risks of participating in this study?**

Regarding the procedure, blood samples for gNBS will be collected from the baby's heel in parallel with the extended newborn metabolic screening.

Therefore, there are some risks associated with the procedure, for example pain for the baby and a small risk of infection; to reduce this risk as much as possible, the midwife will wear gloves and clean the baby's heel before collecting the sample.

Regarding the genetic analysis performed, there is the possibility that the test carried out will be negative only due to a limitation of the technique used (for this reason the quality parameters of the analysis carried out will be reported in the report).

Furthermore, regions of some genes may present characteristics that make analysis problematic: these regions could be partially excluded from the results, as they are difficult to evaluate with sequencing techniques.

The genetic analysis carried out could also identify your child/beneficiary a possible carrier status of recessive genetic diseases (autosomal and X-linked). This data will be communicated to you only upon your request.

Furthermore, there is the possibility, although rare, of experiencing an "unexpected result" (“incidental findings”), for example variants that may reveal information on consanguinity/non-paternity relationships. This information will be communicated to you in compliance with the desire to know/not know the “unexpected results”/“incidental findings”.

Finally, a normal result at newborn genetic screening, through TREAT-panel, does not exclude the presence of a genetic disease not included among those investigated.

- **What are the possible benefits of participating in this study?**

Your child/beneficiary could benefit from early diagnosis of a rare genetic disease for which treatment is available in Italy.

The benefit could be greater if treatment is started in a pre-symptomatic or early phase of the disease; however, we cannot guarantee that treatments will be effective in all cases; furthermore, even with early treatment your child may still show symptoms of the disease.

An early diagnosis could also reduce the burden of multiple diagnostic tests often associated with the “diagnostic odyssey” of a rare genetic disease.

If the newborn genetic screening is negative, you may instead benefit from the reassurance that the presence of one of the rare genetic diseases investigated is very unlikely.

- **Is it possible not to participate or to change my mind?**

Participation in this study is voluntary. You can refuse to allow your child/beneficiary to participate in the study or withdraw from the study at any time, without having to give any explanation and without any penalty or negative consequence. Your refusal to participate or the decision to discontinue participation in the study will in no way influence the care your child/beneficiary receives, which will in any case be the best available.

Physicians can also interrupt the study at any time but must explain the reasons.

Any new information that may influence your decision to continue or not participate in the study will be communicated to you as soon as possible. The same applies to any interruption or suspension of the study.

- **Who to contact for further information and during the study?**

The referring physician for this study is Prof. Alessandra Ferlini, trialsgeneticamedica@unife.it

If you deem it appropriate to report events or facts relating to the trial/study in which you have participated to subjects not directly involved in the trial/study itself, you can refer to the Ethics Committee that approved the trial/study (Ethical Committee of the Vast Emilia Center Area, email comitatoetico@ospfe.it).

- **Collected data**

The data collected will be treated confidentially and anonymously: all data will be coded, and each participant will receive a unique study code. The code, which is the identification number linked to the identity of the participants, will be kept confidentially within the group of researchers involved. Only researchers involved in this study will have access to this code and only they will know the identity of the different participants.
The data collected during this study will be retained for the S4C entire duration.

- **Insurance coverage**

No specific coverage.

- **Biological material**

The biological samples will be stored until the end of the research, unless the interested party consents to storage for a longer period. At the end of the research, the data and samples will be destroyed or made anonymous. At any time you may request the destruction of your child's/beneficiary's biological material.

- **Access to original medical documentation**

Direct access to the original medical documentation of your child/beneficiary will be granted to monitoring or verification personnel, the Ethics Committee and the regulatory authorities for a verification of the study procedures and/or data, without violating your confidentiality, in extent permitted by applicable laws and regulations. By signing the informed consent form, you are authorizing this access. Records identifying your child/beneficiary will be kept confidential and, to the extent permitted by applicable laws and/or regulations, will not be made publicly available.

If the results of the study are published, the identity of your child/beneficiary will remain secret.

Regarding the processing of your child's/beneficiary's personal data, please read related informative form.

- **Information about the results of the study**

The results relating to your child/beneficiary will be communicated to you in the manner described above.

In the event of diagnostic implications of the research data relating to your child/beneficiary, they will be formalized and proposed within the care pathway, therefore with new consents and information forms and always following genetic counselling.

If you are interested and request it, at the end of the trial/study, the general results of the study will be communicated to you.

Furthermore, there may be the possibility that you will be contacted again in the future, for example, to request new information or for new studies (i.e. research purposes) on the material collected.

The protocol of this study and this informative form were drawn up in compliance with the Standards of Good Clinical Practice and the Declaration of Helsinki and were approved by the Ethics Committee of Area Vasta Emilia Centro (CE-AVEC) on…

**CONSENT FORM (on a separate sheet)**

**Title of the study:**
Genetic newborn screening for treatable rare diseases through TREAT-panel within the Screen4Care project

**Local responsible of the study**: Prof.ssa Alessandra Ferlini

Personal data Parent 1

…………………………………………………………………

……………………..……………………….……………………………

……………………………………

telephone…………………….….,

e-mail………………………………………..…………………………..

Personal data Parent 2

…………………………………………………………………

……………………..……………………….……………………………

……………………………………

telephone…………………….….,

e-mail………………………………………..…………………………..

(Name and Surname Child/beneficiary)

…………………………………………………………………

I declare

- that I have received comprehensive explanations regarding my child's/beneficiary to participate in the study, in particular on the purposes and procedures;
- that I had the opportunity to ask questions and have received satisfactory answers;
- that I have read and understood the informative form that was given to me sufficiently in advance;
- that I understand that participation is voluntary, and that my child/beneficiary can be withdrawn from the study at any time, without give explanations and without this influencing his future assistance in any way;
- to be aware that, if I withdraw my consent, the data of my child/beneficiary collected before the withdrawal of consent will be used by the researcher;
- to have received the informative form regarding genetic investigations.

Consequently to these statements:

- **I accept** that my child/beneficiary will participate in the study

  NO YES
- I **accept** to have my child/beneficiary undergo to any further diagnostic tests for the completeness of the test

NO YES

- **I accept** to be informed about the results obtained and their meaning, aware of the family implications relating to the test to which I consent

NO YES

- **I accept** to be informed about the identification of my child/beneficiary as a carrier of recessive pathologies (autosomal and X-linked)

NO YES

- **I accept** to be contacted in the future for further genetic analyses (whole genome sequencing, WGS) in the event of the onset of early symptoms suggestive of a genetic disease or for new studies on the material collected

NO YES

- **I accept** to be contacted in the future to provide new information or for new studies (i.e. for research purposes) on the material collected

NO YES

- **I accept** that the results acquired through carrying out investigations using the samples and the data associated are used anonymously for scientific publications, statistics and scientific conferences

NO YES

- **I accept** that the Paediatrician will be informed of the participation of my child/beneficiary in the study

NO YES

Name and Surname Parent 1

………………………………………………………………

Date……………………………..

Signature……………………………………………………………………

Name and Surname Parent 2

…………………………………………………………………….

Date……………………………..

Signature………………………………………………………………………

Name of the person collecting informed consent

………………………………….

Date……………………………

Signature………………………………………………………………………

Email: trialsgeneticamedica@unife.it

Notes: 1 copy for the participant, 1 copy for the study manager and 1 copy to keep in the medical record.
