# Supplementary material for: European Reference Networks as core health structures where referring genetic newborn screening positive infants: an innovative operational research framework
Source: Front Public Health. 2026 Jun 10;14:1822461. doi: 10.3389/fpubh.2026.1822461 (PMC13292599; doi:10.3389/fpubh.2026.1822461)
Supplement: Supplementary file 3 [file Data_Sheet_3.pdf]

REPORT OF THE TREAT PANEL RESULTS WITHIN THE SCREEN4CARE RESEARCH PROJECT

Dear Parents,

as stated in the study's informed consent form (approved on XXX), which specifies that parents of children with negative results will receive written notification of the outcome, we hereby send you this letter.

In reference to the consent, you provided for participation in the Screen4Care research project, funded by the European Community (www.screen4care.eu), we inform you that the analysis of the TREAT-panel (*Orphanet J Rare Dis. 2025 May 15;20(1):231*), which includes 245 genes involved in rare genetic diseases, did not identify any pathogenic or likely pathogenic variants in the genes analyzed, thus yielding a negative result for the presence of pathological genotypes in your child.

Notification of any carrier status in the child (if permitted), which does not imply any disease or susceptibility to known conditions, may be provided upon completion of the project.

Should any updates concerning your child's health arise, we kindly invite you to contact us at the following email address: trialsgeneticamedica@unife.it

We remain at your disposal for any further clarification.

The PI of the S4C UNIFE Team

Prof. Alessandra Ferlini

trialsgeneticamedica@unife.it

This project has received funding from the Innovative Medicines Initiative 2 Joint Undertaking (JU) under grant agreement No. 101034427. The JU receives support from the European Union's Horizon 2020 research and innovation programme and EFPIA. www.imi.europa.eu
