# Supplementary material for: European Reference Networks as core health structures where referring genetic newborn screening positive infants: an innovative operational research framework
Source: Front Public Health. 2026 Jun 10;14:1822461. doi: 10.3389/fpubh.2026.1822461 (PMC13292599; doi:10.3389/fpubh.2026.1822461)
Supplement: Supplementary file 5 [file Data_Sheet_5.pdf]

FAQ

1) What are genetic diseases?

Rare diseases are genetic in origin (about 85%) and are caused by the presence of alterations ("errors") in DNA.

The genetic heritage of each of us is written in our DNA sequence, which is contained in the nucleus of our body cells and is packaged in particular structures called chromosomes. These chromosomes contain **genes**, which are specific portions that are used to make the proteins we are made of.

Monogenic hereditary diseases are caused by mutations in a single gene; examples include Mediterranean anemia, cystic fibrosis, and Duchenne muscular dystrophy. Monogenic diseases are studied with **molecular genetic analyses**.

2) How frequent are genetic diseases?

Genetic diseases are rare… but not *that* rare! In fact, there are **more than 7,000** of them.
Each genetic disease is rare (found in less than 1 in 2000 people), but overall they affect **1 in 17 people**. They involve **36 million people** in the European Union.
Most rare diseases have a **pediatric onset**.
The average time to reach a diagnosis is 5 **years**!
The goal of the Screen4Care Project is to accelerate the diagnosis of rare diseases through neonatal genetic screening which means screening babies shortly after birth for the early identification of some monogenic diseases.

3) Which newborn (metabolic) screening is currently available for all newborns in Italy?

Extended newborn screening is currently available and mandatory in Italy: it checks for more than 40 diseases (genetic, endocrine and metabolic) which, if not recognized early, could negatively affect the child's development. Italy is first in Europe for the number of diseases included in the screening.

Within 48-72 hours of birth, all babies are given a small puncture in the heel in order to collect a few drops of blood on a special **absorbent card (DBS)**, which allows a simple screening test to be carried out at a laboratory of the region's reference screening center.

4) What is the European Screen4Care Project? What does it propose?

The Screen4Care Research Project, created in 2021, is a European Consortium, which will last 5 years. The Screen4Care project involves universities and pharmaceutical companies from all over Europe as well as

EURORDIS, the umbrella patient association for rare diseases. The Scientific Coordinator of the Project is the the University of Ferrara. The aim of the Project is to shorten the time to the diagnosis of rare genetic diseases through the use of **digital technologies** and **genetic newborn screening**.

5) <u>What is the difference between metabolic newborn screening and genetic newborn screening?</u>

Metabolic newborn screening is performed at birth by taking drops of blood from the newborn's heel. This screening allows us to identify some of the known rare diseases by analyzing certain substances in the blood (called **metabolites**). It is not primarily a genetic analysis, but rather a technique that permits the effective identification of certain metabolic diseases.

The **SCREEN4CARE** (S4C) Project offers an innovative research approach through **newborn genetic screening (gNBS)**, which consists in the initial analysis of some DNA **genes** on blood collected from an additional dried blood card sample. Genetic screening will be offered (**in addition** to newborn screening, not as a replacement!) to a maximum number of 25 thousand newborns in Europe and will allow the identification of monogenic diseases that may not be evident at birth or that are not identifiable with metabolic newborn screening.

6) <u>On which newborn blood sample will the newborn genetic screening of the S4C Project be performed?</u>

The sample will be collected at the same time as the small heel prick for metabolic screening: instead of one, two absorbent cards (**DBS**) will be collected. One will be dedicated to metabolic screening, the other to genetic screening for the Screen4Care project. The additional sample will be delivered to the Genetics Laboratory of the S4C Project. Here, DNA will be extracted from the blood in order to perform genetic analyses for neonatal genetic screening.

7) <u>Which diseases does the newborn genetic screening of the Screen4Care Project analyze?</u>

Newborn genetic screening in the S4C Project will screen RDs where the genetic case is known, when the genetic alterations is identifyible and for diseases where there is a treatment available . We will use a gene panel , called TREAT, which includes all the selected genes for diseases where a treatment is available.

These diseases are of various types: metabolic, neuromuscular, endocrinological, immunological and so on. Please see in the figure below the types of diseases we will screen depending on their clinical characteristics. The European Reference Network, the excellence centers for rare diseases in Europe, will be in charge for the follow up of that specific diseases, and are detailed below.

If you would like to receive a complete list of the diseases that we screen for in the S4C project, you can contact Doctors at the Project email:

trialsgeneticamedica@unife.it (UNIFE birth site)

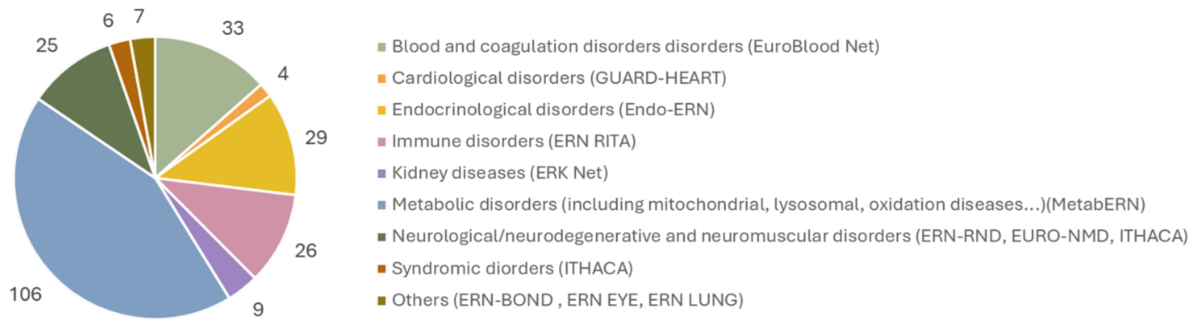

8) In addition to newborn genetic screening, does the project offer anything else?

Yes, for newborns that test negative at the newborn genetic screening but show early symptoms in the first one to two years of life, which might be even not related to the genes screened by the TREAT panel, Screen4Care offers **Whole Genome Sequencing (WGS). WGS will explore a large set of gene (called Mendeliome) to provide a defined diagnosis to the symptomatic babies.** Indeed, this is the analysis of several genes (about 4000) so many more than those explored in the TREAT panel.

9) How can I join the project? Are there any costs to join?

In the Screen4Care Project, you can choose between:

- participate to the TREAT panel at birth only

-participate to both TREAT panel and Whole Genome Sequences (only in case of early symptoms)

In short, you can join:

• Newboorn genetic screening using the TREAT panel
• Newborn genetic screening using the TREAT panel and early diagnostic screening by WGS

…or not join the Screen4Care Project at all.

The choice to join is voluntary and free. If you decide to join the S4C project, both child's parents will be asked to sign a written informed form.

We will meet you during the pregnancy and after the birth to explain the project, show you videos, collect informed consent forms and answer your questions.

10) What results can I expect from genetic newborn screening (TREAT-panel)?

Screening can give 2 results: negative or positive.

If the gNBS result is **negative**, it means that no alterations were found in the analysed genes . In that case, parents will not be contacted but will receive a letter attesting the negative test result. If you do not receive the letter within 3 months of birth, please contact us at trialsgeneticamedica@unife.it.

If the gNBS result is **positive**,this means that your child may have a genetic disease and needs further evalulation. Then, you be contacted for an interview, and will be referred to a center belonging to European Centers of Excellence network (ERN) for clinical and diagnostic confirmation, genetic counseling to discuss the implications of the results for the family, clinical follow-up and access to therapies.

In some cases, an uncertain result may occur and further investigation may be necessary.

We are committed to provide newborn screening results in about 120 days, while WGS output will follow ERN-specific and disease-specific turnaround reporting time.

11) Why join theScreen4Care project?

The benefits of the TREAT-panel are the possibility of screening for an early diagnosis, which allows access to reference centers for available therapies and treatments, as well as the possibility of receive genetic counselling .

WGS allows for the timely diagnosis of a genetic disease in the event of symptoms and when the TREAT panel has resulted negative. Like for the TREAT-panel, access to Rare Diseases excellence centers (European Reference Networks) will be timely granted to get access of the full cohort of standard of care, and, possibly, therapy or clinical trials access.

12) What are the risks of joining the TREAT-panel and/or the WGS?

Concerning risk factors, as with any blood sample, there is a minimal risk associated with the heel blood sample for the TREAT panel, which is in any case required by law for metabolic screening. Fresh blood taken will be necessary for WGS , therefore a minimal invasive procedure .

Furthermore, since these are genetic analyses, further results may emerge not concerning the baby's health only but also carrier status in parents . These could include that one of the parent, even if healthy, carries the alterations in the analyzed gene, so called healthy carrier.

WGS may disclose incidental or unexpected results or secondary findings (such as consanguinity or non-paternity). Non paternity will not be communicated, while carrier status might be communicated if agreed in the informed consent, and might vary in S4C countries.

Finally, the possible emotional implications of an early diagnosis of a genetic disease in your child will need to be considered.

13) <u>Where will the genetic analysis of the TREAT-panel be performed?</u>

The additional dried blood spot sample will be sent to the Medical Genetics Laboratory of the Bambino Gesù Pediatric Hospital in Rome. The analysis of the test will then be performed by partners of the Screen4Care Project. The result of the TREAT-panel will be available within 90 days by directly contacting the couple by letter, telephone, or email and proposing an in person interview, also the child's sample will be kept for three years.

14) <u>Do I have to join the project?</u>

Certainly not! If you decide not to join the Screen4care Project, only the metabolic screening will be performed on newborns as scheduled by the national screening.

Remember that joining the Screen4Care Project **DOES NOT REPLACE** the checks required during pregnancy and extended newborn screening.

15) <u>In summary…</u>

You can join:

- neonatal genetic screening using the TREAT panel in order to identify treatable diseases for which there is a therapy
- neonatal genetic screening using the TREAT panel <u>and</u> WGS within the first one to two years of life

…or **not join** the Screen4Care Project at all.

Participation is **voluntary**, not mandatory, and must be expressed through **written informed consent for each of the options chosen**.

Participation is **free**.

Participation to the Screen4Care Project **DOES NOT REPLACE** the checks required during pregnancy and extended newborn screening.

16) <u>Contacts</u>

You can follow us on our social networks and ask for all the information you want!

Connect with Screen4Care at:

- https://www.screen4care.eu/ (available in English, Italian, German and French)
- https://twitter.com/screen4care
- https://www.linkedin.com/company/screen4care/
- https://www.youtube.com/channel/screen4care

Contacts in Italy:

- Ferrara → trialsgeneticamedica@unife.it
