# Supplementary material for: European Reference Networks as core health structures where referring genetic newborn screening positive infants: an innovative operational research framework
Source: Front Public Health. 2026 Jun 10;14:1822461. doi: 10.3389/fpubh.2026.1822461 (PMC13292599; doi:10.3389/fpubh.2026.1822461)
Supplement: Supplementary file 6 [file Data_Sheet_6.pdf]

Ferrara, 22 November 2025

Dear ERN Coordinator,

As you know the **Screen4Care research project** (S4C, www.screen4care.eu) funded by the European Union, aims at reducing the time to diagnosis and treatment of rare diseases through genetic newborn screening (gNBS).
This screening is performed using an NGS panel that includes 245 genes associated with treatable conditions, known as “TREAT panel.”
The very important and fruitful S4C-ERNs meetings we have had in the last 3 years, have allowed us the drafting of **S4C-ERN framework operational guidelines**, which have been shared and finalized and will be submitted to the PlosOne journal as “Clinical Protocol” article. In this document we agreed that newborns who test positive at the TREAT panel will be referred to the appropriate ERN HCP for diagnostic work-up, multidisciplinary post-test follow-up, and access to available therapies, as being this integral part of the ERNs mission.
According to the pipeline, S4C will provide the couple with positive babies with a validated report which should be consolidated by genotype-phenotype correlation and standard of care, done by ERNs. Diagnostic work-up and eventual minimal patients’-related costs will be eventually covered according to the rule of the specific HCP.

We would appreciate if you can circulate by email this letter among all your ERN HCPs, both to provide S4C contacts and to correctly inform them about this collaborative ERN-S4C framework.

We would also kindly ask you to write to your HCPs a letter encouraging them to support and participate to the ERN-S4C framework to maximize the benefit of the S4C newborn screening output aiming at providing an early diagnosis to babies affected with rare diseases and therefore to optimize and accelerate their diagnostic work-up and early access to therapies

Thank you in advance for your valuable cooperation.

Best Regards,
Alessandra Ferlini, Screen4Care project Scientific Coordinator

Contact Emails:
screen4care@unife.it
trialsgeneticamedica@unife.it
Fernanda Fortunato, Study Coordinator TREAT panel trial
(ClinicalTrials.gov ID NCT06549218)

Below: S4C-ERN pipeline

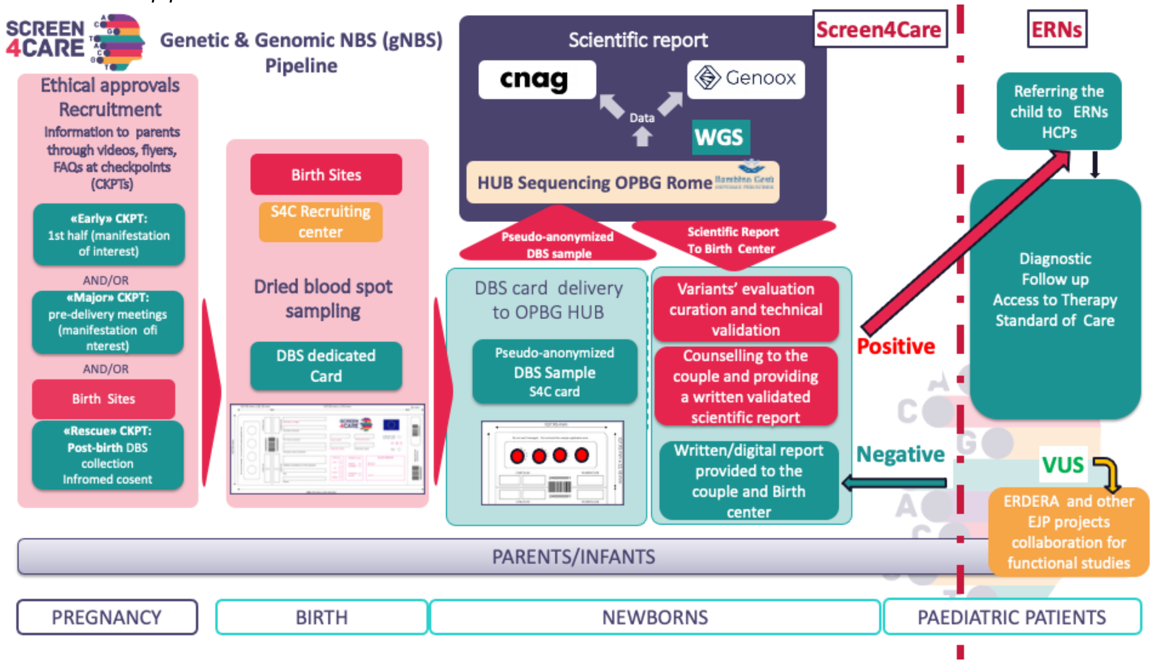

Università degli Studi di Ferrara

This project has received funding from the Innovative Medicines Initiative 2 Joint Undertaking (JU) under grant agreement No 101034427.
The JU receives support from the European Union’s Horizon 2020 research and innovation programme and EFPIA.
