# Supplementary material for: European Reference Networks as core health structures where referring genetic newborn screening positive infants: an innovative operational research framework
Source: Front Public Health. 2026 Jun 10;14:1822461. doi: 10.3389/fpubh.2026.1822461 (PMC13292599; doi:10.3389/fpubh.2026.1822461)
Supplement: Supplementary file 7 [file Presentation_1.pptx]

## Slide 1
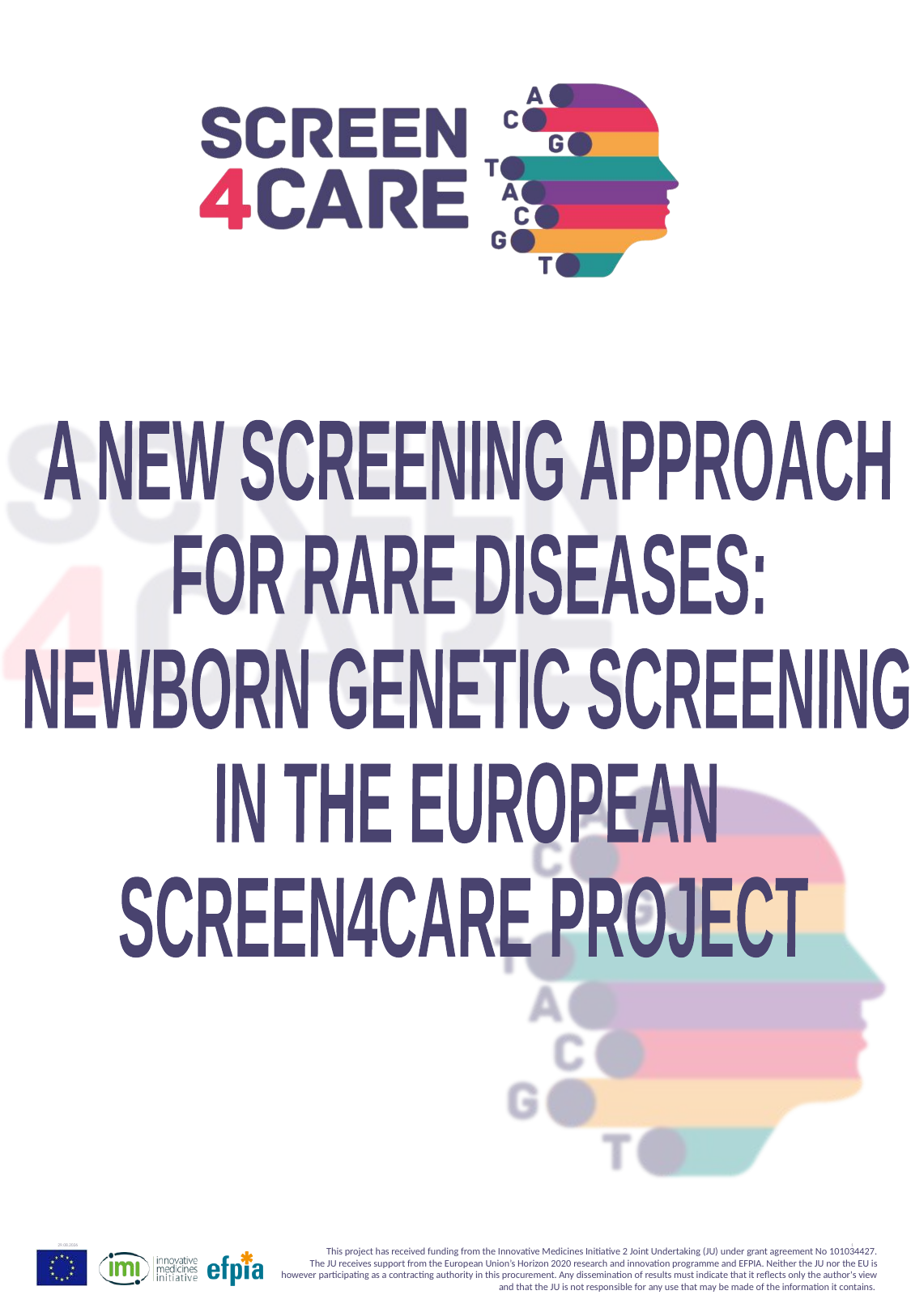

A NEW SCREENING APPROACH
FOR RARE DISEASES:
NEWBORN GENETIC SCREENING
IN THE EUROPEAN
SCREEN4CARE PROJECT
06.12.2025
1
This project has received funding from the Innovative Medicines Initiative 2 Joint Undertaking (JU) under grant agreement No 101034427.
The JU receives support from the European Union’s Horizon 2020 research and innovation programme and EFPIA. Neither the JU nor the EU is however participating as a contracting authority in this procurement. Any dissemination of results must indicate that it reflects only the author's view and that the JU is not responsible for any use that may be made of the information it contains.

## Slide 2
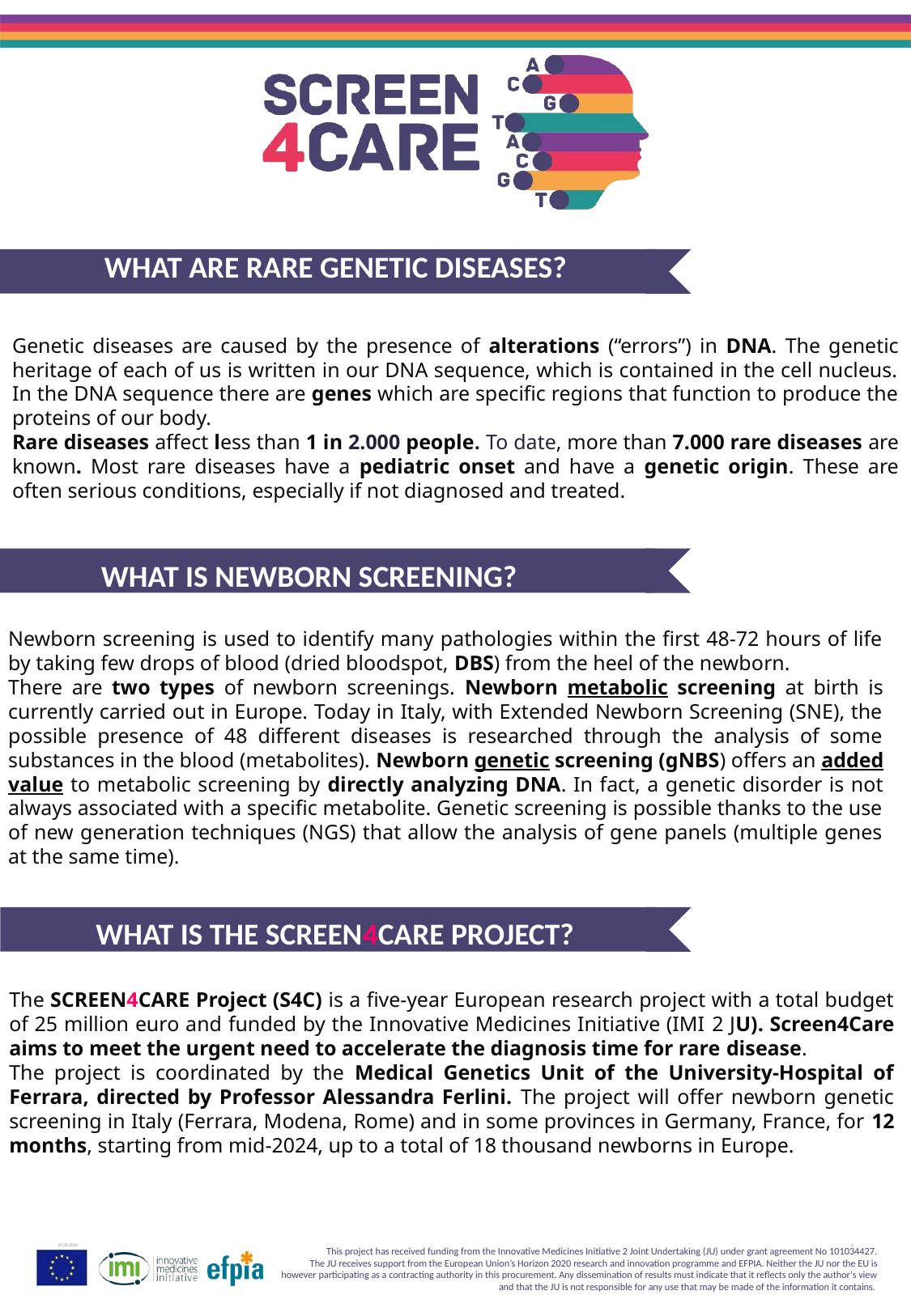

WHAT ARE RARE GENETIC DISEASES?
Genetic diseases are caused by the presence of alterations (“errors”) in DNA. The genetic heritage of each of us is written in our DNA sequence, which is contained in the cell nucleus. In the DNA sequence there are genes which are specific regions that function to produce the proteins of our body.
Rare diseases affect less than 1 in 2.000 people. To date, more than 7.000 rare diseases are known. Most rare diseases have a pediatric onset and have a genetic origin. These are often serious conditions, especially if not diagnosed and treated.
WHAT IS NEWBORN SCREENING?
Newborn screening is used to identify many pathologies within the first 48-72 hours of life by taking few drops of blood (dried bloodspot, DBS) from the heel of the newborn.
There are two types of newborn screenings. Newborn metabolic screening at birth is currently carried out in Europe. Today in Italy, with Extended Newborn Screening (SNE), the possible presence of 48 different diseases is researched through the analysis of some substances in the blood (metabolites). Newborn genetic screening (gNBS) offers an added value to metabolic screening by directly analyzing DNA. In fact, a genetic disorder is not always associated with a specific metabolite. Genetic screening is possible thanks to the use of new generation techniques (NGS) that allow the analysis of gene panels (multiple genes at the same time).
WHAT IS THE SCREEN4CARE PROJECT?
The SCREEN4CARE Project (S4C) is a five-year European research project with a total budget of 25 million euro and funded by the Innovative Medicines Initiative (IMI 2 JU). Screen4Care aims to meet the urgent need to accelerate the diagnosis time for rare disease.
The project is coordinated by the Medical Genetics Unit of the University-Hospital of Ferrara, directed by Professor Alessandra Ferlini. The project will offer newborn genetic screening in Italy (Ferrara, Modena, Rome) and in some provinces in Germany, France, for 12 months, starting from mid-2024, up to a total of 18 thousand newborns in Europe.
12/6/2025
2
This project has received funding from the Innovative Medicines Initiative 2 Joint Undertaking (JU) under grant agreement No 101034427.
The JU receives support from the European Union’s Horizon 2020 research and innovation programme and EFPIA. Neither the JU nor the EU is however participating as a contracting authority in this procurement. Any dissemination of results must indicate that it reflects only the author's view and that the JU is not responsible for any use that may be made of the information it contains.

## Slide 3
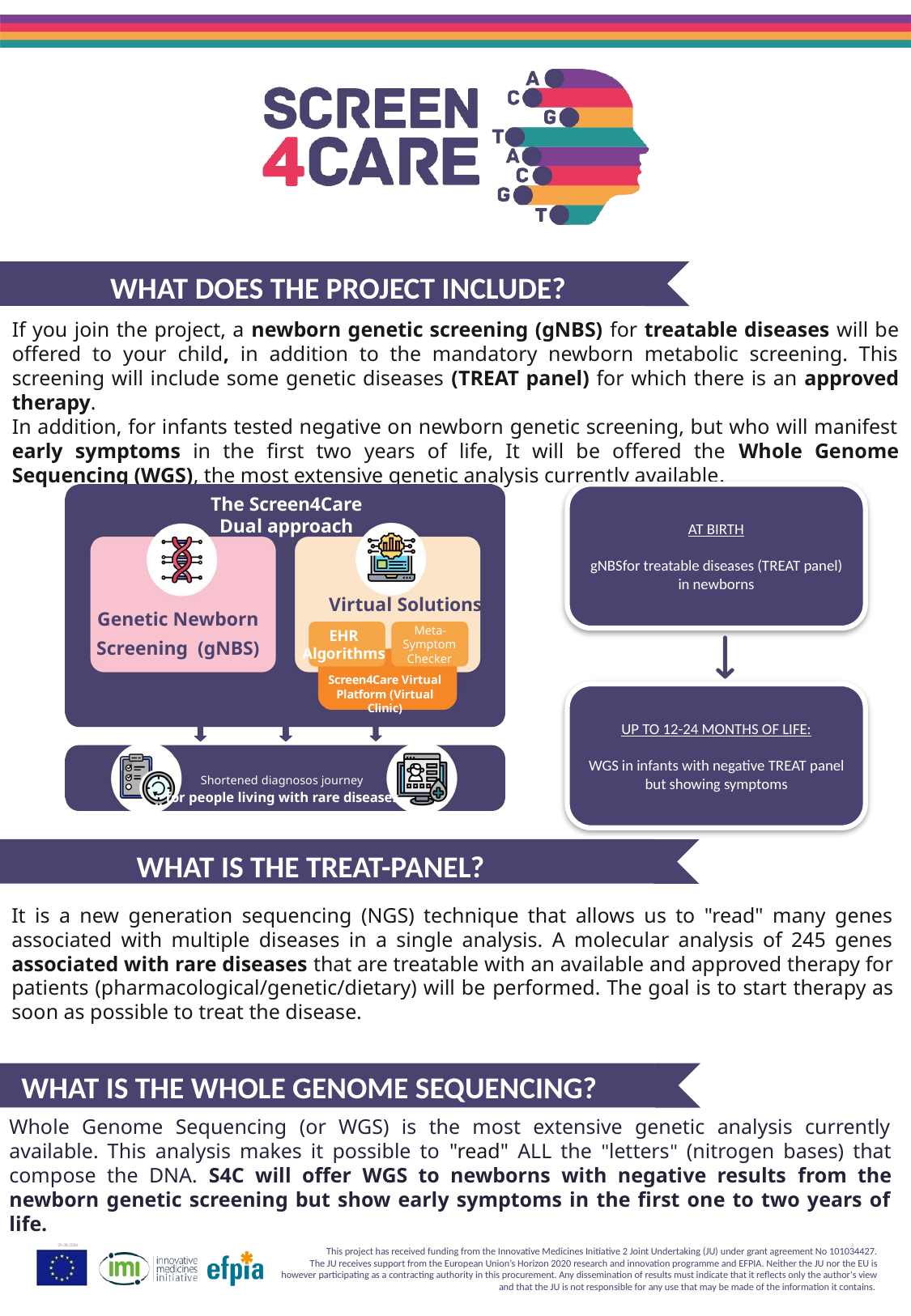

WHAT DOES THE PROJECT INCLUDE?
If you join the project, a newborn genetic screening (gNBS) for treatable diseases will be offered to your child, in addition to the mandatory newborn metabolic screening. This screening will include some genetic diseases (TREAT panel) for which there is an approved therapy.
In addition, for infants tested negative on newborn genetic screening, but who will manifest early symptoms in the first two years of life, It will be offered the Whole Genome Sequencing (WGS), the most extensive genetic analysis currently available.
AT BIRTH
gNBSfor treatable diseases (TREAT panel) in newborns
UP TO 12-24 MONTHS OF LIFE:
WGS in infants with negative TREAT panel but showing symptoms
The Screen4Care Dual approach
Virtual Solutions
Genetic Newborn
Screening (gNBS)
Meta-
Symptom Checker
EHR
Algorithms
Screen4Care Virtual Platform (Virtual Clinic)
Shortened diagnosos journey
for people living with rare diseases
WHAT IS THE TREAT-PANEL?
It is a new generation sequencing (NGS) technique that allows us to "read" many genes associated with multiple diseases in a single analysis. A molecular analysis of 245 genes associated with rare diseases that are treatable with an available and approved therapy for patients (pharmacological/genetic/dietary) will be performed. The goal is to start therapy as soon as possible to treat the disease.
WHAT IS THE WHOLE GENOME SEQUENCING?
Whole Genome Sequencing (or WGS) is the most extensive genetic analysis currently available. This analysis makes it possible to "read" ALL the "letters" (nitrogen bases) that compose the DNA. S4C will offer WGS to newborns with negative results from the newborn genetic screening but show early symptoms in the first one to two years of life.
06.12.2025
3
This project has received funding from the Innovative Medicines Initiative 2 Joint Undertaking (JU) under grant agreement No 101034427.
The JU receives support from the European Union’s Horizon 2020 research and innovation programme and EFPIA. Neither the JU nor the EU is however participating as a contracting authority in this procurement. Any dissemination of results must indicate that it reflects only the author's view and that the JU is not responsible for any use that may be made of the information it contains.

## Slide 4
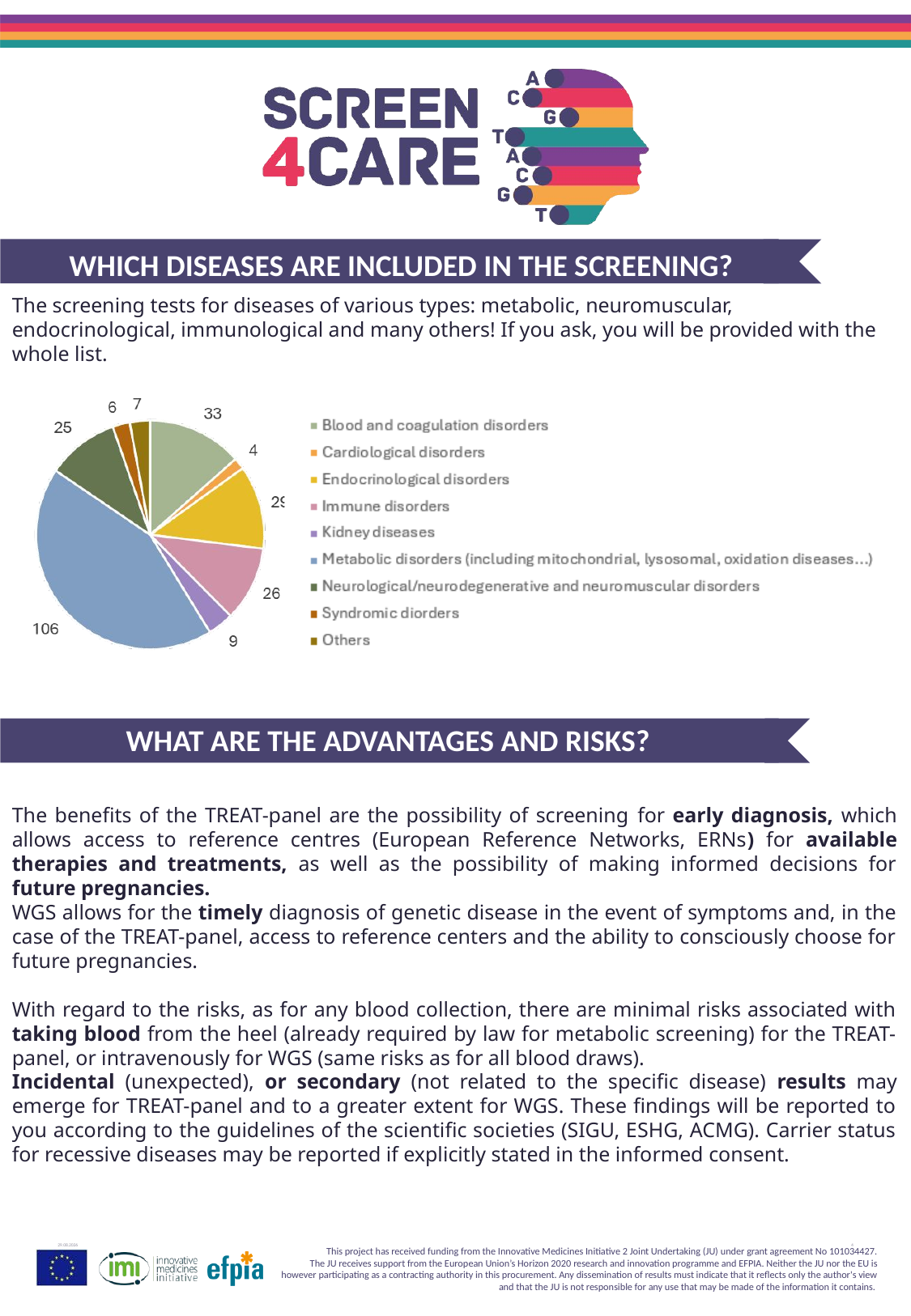

WHICH DISEASES ARE INCLUDED IN THE SCREENING?
The screening tests for diseases of various types: metabolic, neuromuscular, endocrinological, immunological and many others! If you ask, you will be provided with the whole list.
WHAT ARE THE ADVANTAGES AND RISKS?
The benefits of the TREAT-panel are the possibility of screening for early diagnosis, which allows access to reference centres (European Reference Networks, ERNs) for available therapies and treatments, as well as the possibility of making informed decisions for future pregnancies.
WGS allows for the timely diagnosis of genetic disease in the event of symptoms and, in the case of the TREAT-panel, access to reference centers and the ability to consciously choose for future pregnancies.
With regard to the risks, as for any blood collection, there are minimal risks associated with taking blood from the heel (already required by law for metabolic screening) for the TREAT-panel, or intravenously for WGS (same risks as for all blood draws).
Incidental (unexpected), or secondary (not related to the specific disease) results may emerge for TREAT-panel and to a greater extent for WGS. These findings will be reported to you according to the guidelines of the scientific societies (SIGU, ESHG, ACMG). Carrier status for recessive diseases may be reported if explicitly stated in the informed consent.
06.12.2025
4
This project has received funding from the Innovative Medicines Initiative 2 Joint Undertaking (JU) under grant agreement No 101034427.
The JU receives support from the European Union’s Horizon 2020 research and innovation programme and EFPIA. Neither the JU nor the EU is however participating as a contracting authority in this procurement. Any dissemination of results must indicate that it reflects only the author's view and that the JU is not responsible for any use that may be made of the information it contains.

## Slide 5
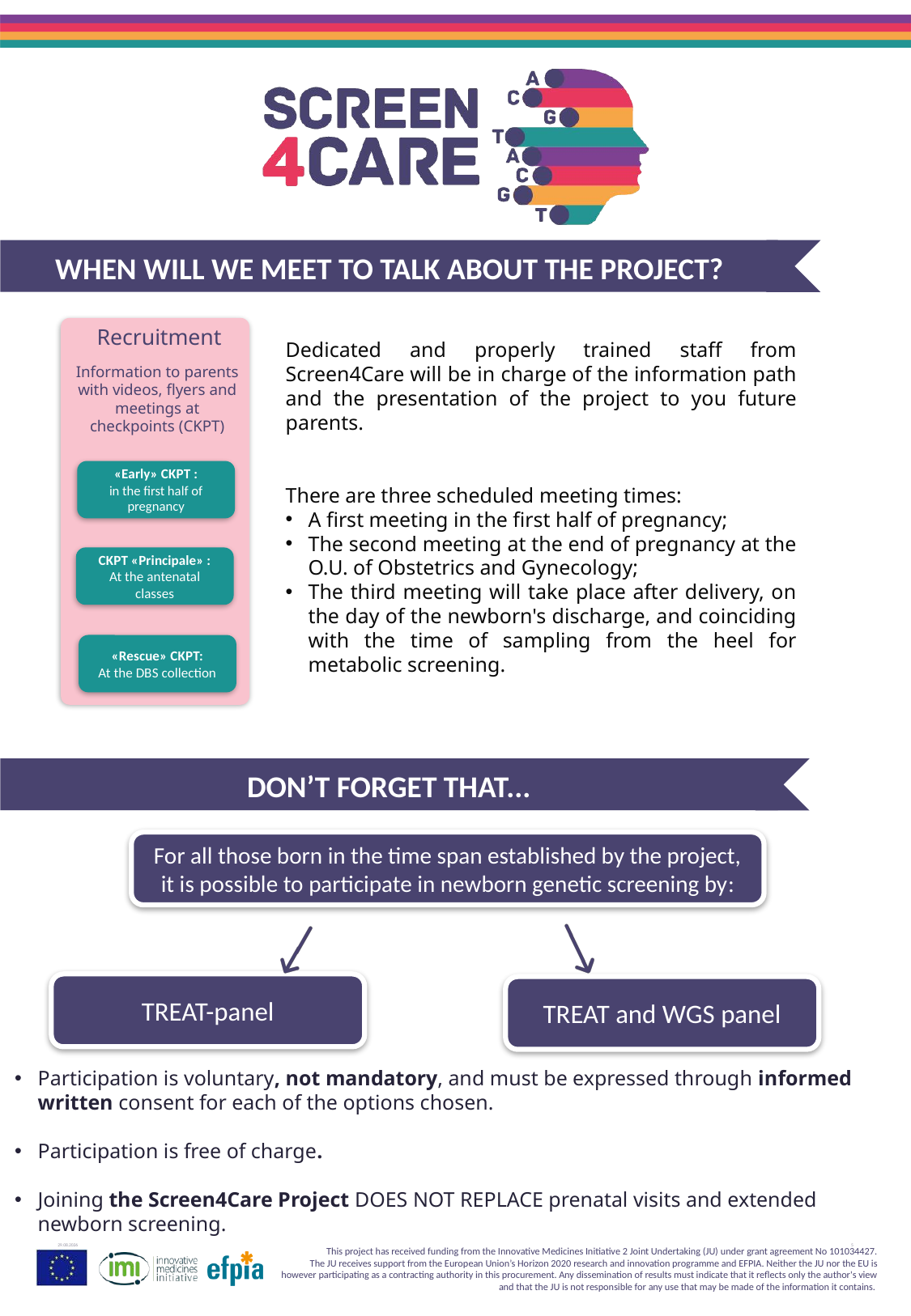

WHEN WILL WE MEET TO TALK ABOUT THE PROJECT?
Recruitment
Dedicated and properly trained staff from Screen4Care will be in charge of the information path and the presentation of the project to you future parents.
There are three scheduled meeting times:
A first meeting in the first half of pregnancy;
The second meeting at the end of pregnancy at the O.U. of Obstetrics and Gynecology;
The third meeting will take place after delivery, on the day of the newborn's discharge, and coinciding with the time of sampling from the heel for metabolic screening.
Information to parents with videos, flyers and meetings at checkpoints (CKPT)
«Early» CKPT :
in the first half of pregnancy
CKPT «Principale» :
At the antenatal classes
«Rescue» CKPT:
At the DBS collection
DON’T FORGET THAT...
For all those born in the time span established by the project, it is possible to participate in newborn genetic screening by:
TREAT-panel
TREAT and WGS panel
Participation is voluntary, not mandatory, and must be expressed through informed written consent for each of the options chosen.
Participation is free of charge.
Joining the Screen4Care Project DOES NOT REPLACE prenatal visits and extended newborn screening.
06.12.2025
5
This project has received funding from the Innovative Medicines Initiative 2 Joint Undertaking (JU) under grant agreement No 101034427.
The JU receives support from the European Union’s Horizon 2020 research and innovation programme and EFPIA. Neither the JU nor the EU is however participating as a contracting authority in this procurement. Any dissemination of results must indicate that it reflects only the author's view and that the JU is not responsible for any use that may be made of the information it contains.

## Slide 6
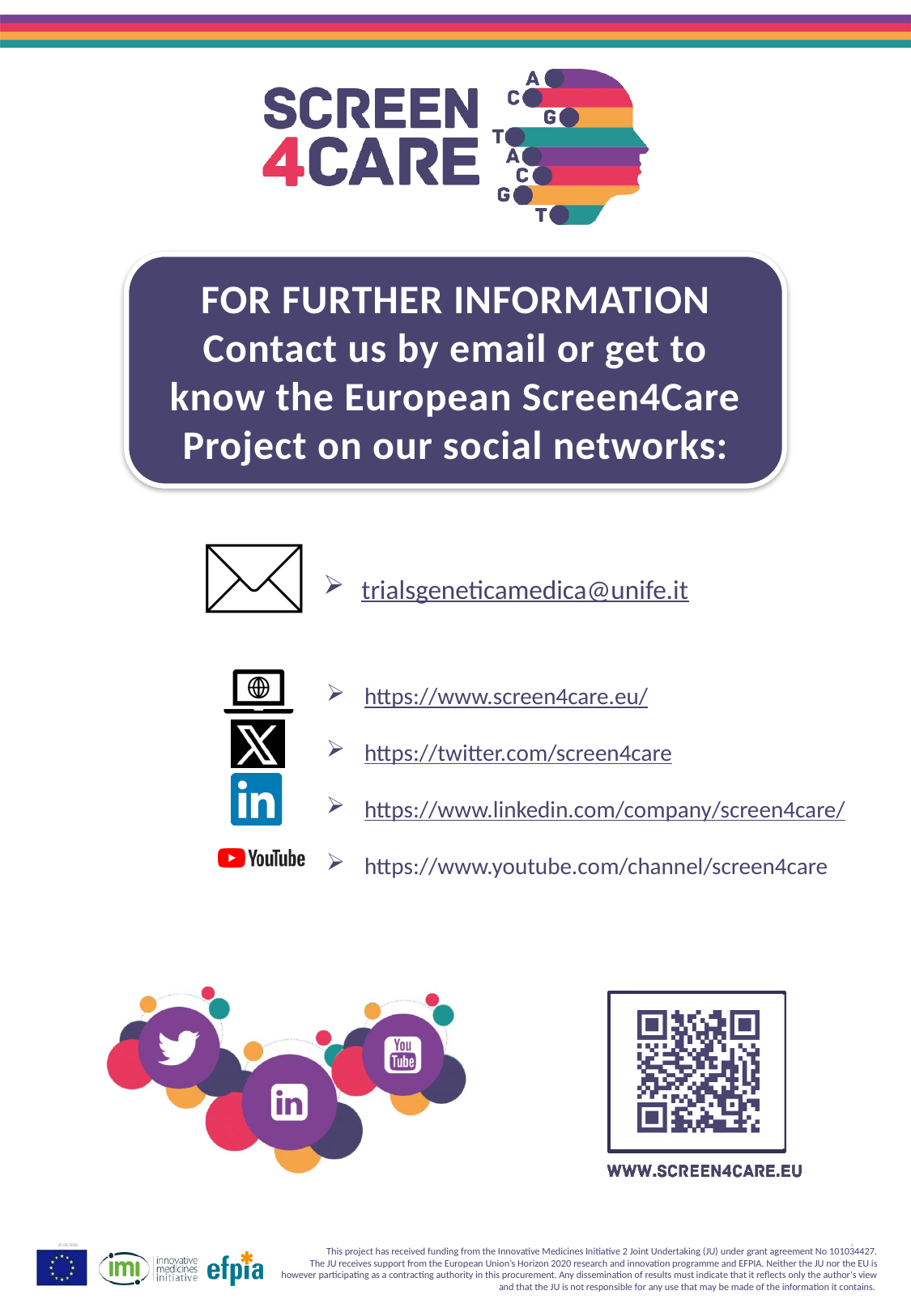

FOR FURTHER INFORMATION
Contact us by email or get to know the European Screen4Care Project on our social networks:
trialsgeneticamedica@unife.it
https://www.screen4care.eu/
https://twitter.com/screen4care
https://www.linkedin.com/company/screen4care/
https://www.youtube.com/channel/screen4care
06.12.2025
6
This project has received funding from the Innovative Medicines Initiative 2 Joint Undertaking (JU) under grant agreement No 101034427.
The JU receives support from the European Union’s Horizon 2020 research and innovation programme and EFPIA. Neither the JU nor the EU is however participating as a contracting authority in this procurement. Any dissemination of results must indicate that it reflects only the author's view and that the JU is not responsible for any use that may be made of the information it contains.
