# Supplementary figures and images for: European Reference Networks as core health structures where referring genetic newborn screening positive infants: an innovative operational research framework
Source: Front Public Health. 2026 Jun 10;14:1822461. doi: 10.3389/fpubh.2026.1822461 (PMC13292599; doi:10.3389/fpubh.2026.1822461)

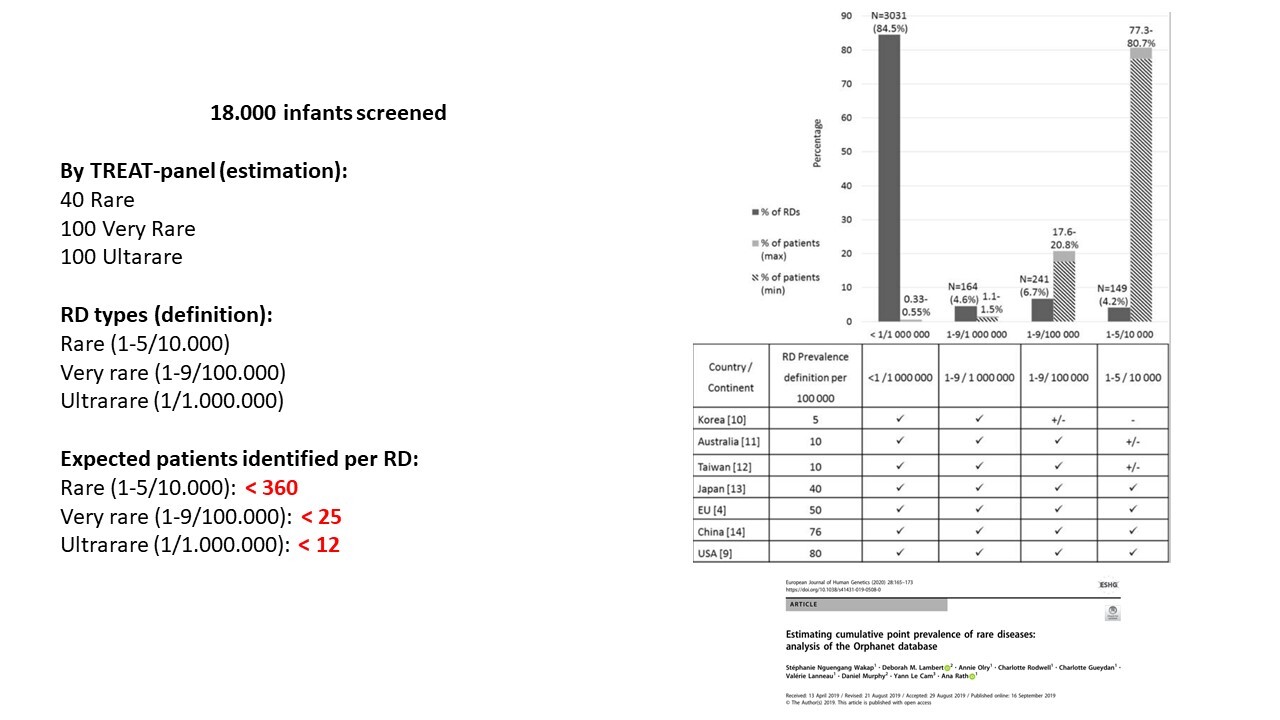

Supplement: Supplementary file 8 [file Image_1.jpeg]
