# Supplementary material for: European Reference Networks as core health structures where referring genetic newborn screening positive infants: an innovative operational research framework
Source: Front Public Health. 2026 Jun 10;14:1822461. doi: 10.3389/fpubh.2026.1822461 (PMC13292599; doi:10.3389/fpubh.2026.1822461)
Supplement: Supplementary file 4 [file Data_Sheet_4.pdf]

CURATED GENETIC RESEARCH REPORT ABOUT SCREEN4CARE TREAT PANEL TOOL

S4C EU Birth site: XXX

Contact person: XXX

Dear Dr XXX,

Based on the informed consent Parents (ID XX) and (ID YY) have signed the (date of the signed informed consent) to participate to the research project Screen4Care (screen4care.eu), funded by the European Union and by EFPIA consortium, please find below the results we obtained from the piloting of the TREAT panel tool (Orphanet J Rare Dis. 2025 May 15;20(1):231) which includes 245 genes involved in treatable rare diseases, on your enrolled babies, as registered in ClinicalTrial.Gov N. NCT06549218.

Your baby (XXX) born (DOB) at (Birth Site) resulted positive for the following genotype:

Birth Site	Sex	ID	Result	Gene	Type of Variant	Annotation	Inheritance	Curated Result

Gene:<br>Genotype:<br>Variant:<br>Associated Rare Disease: OMIM #<br>Inheritance:<br><br>Validation (click as applicable):<br>A) The panel results are considered validated<br>B) The variant (XX) has been technically validated using the following method (MLPA/RealTime)

Based on the identified genotype and the heterozygous variant we recommend:

-segregation analysis

-diagnostic grade genetic validation (on a novel fresh biological sample from the baby)

-eventual clinical follow up

To get access to a clinical pathway fully compliant with diagnostic and health procedures we suggest

This project has received funding from the Innovative Medicines Initiative 2 Joint Undertaking (JU) under grant agreement No. 101034427. The JU receives support from the European Union's Horizon 2020 research and innovation programme and EFPIA. www.imi.europa.eu

to contact the following European Reference Network (ERN):

to carry on in a clinical and diagnostic setting the procedures mentioned above, to evaluate a metabolic confirmation, in case a specific biomarker might be available, to set up all eventual further tests which might be necessary to confirm the genotype/phenotype, and the related follow up including possible treatments, in case the diagnosis will be fully confirmed.

The UNIFE PI of S4C TEAM

Prof.ssa Alessandra Ferlini

trialsgeneticamedica@unife.it

This project has received funding from the Innovative Medicines Initiative 2 Joint Undertaking (JU) under grant agreement No. 101034427. The JU receives support from the European Union's Horizon 2020 research and innovation programme and EFPIA. www.imi.europa.eu
